# Epigenome-Wide and Methylation Risk Score Analysis of Body Mass Index Among People with HIV

**DOI:** 10.3390/epigenomes8040046

**Published:** 2024-12-12

**Authors:** Nanzha Abi, Alexandra Young, Pradeep Tiwari, Junyu Chen, Chang Liu, Qin Hui, Kaku So-Armah, Matthew S. Freiberg, Amy C. Justice, Ke Xu, Marta Gwinn, Vincent C. Marconi, Yan V. Sun

**Affiliations:** 1 Department of Epidemiology, Rollins School of Public Health, Emory University, Atlanta, GA 30322, USA; nanzha.abi@emory.edu (N.A.); alexandra.young@emory.edu (A.Y.); pradeep.tiwari@emory.edu (P.T.); junyu.chen@emory.edu (J.C.); chang.liu2@emory.edu (C.L.); qhui@emory.edu (Q.H.); mlg1@cdc.gov (M.G.); 2 Atlanta Veterans Affairs Health Care System, Decatur, GA 30033, USA; 3 Boston University School of Medicine, Boston, MA 02118, USA; kaku@bu.edu; 4 Cardiovascular Medicine Division, Vanderbilt University School of Medicine and Tennessee Valley Healthcare System, Nashville, TN 37212, USA; matthew.s.freiberg@vumc.org; 5 Connecticut Veteran Health System, West Haven, CT 06516, USA; amy.justice2@va.gov (A.C.J.); ke.xu@yale.edu (K.X.); 6 Schools of Medicine and Public Health, Yale University, New Haven, CT 06520, USA; 7 Department of Psychiatry, Yale School of Medicine, New Haven, CT 06520, USA; 8 Hubert Department of Global Health, Rollins School of Public Health, Emory University, Atlanta, GA 30322, USA; 9 Division of Infectious Diseases, Emory University School of Medicine, Atlanta, GA 30322, USA

**Keywords:** body mass index, EWAS, HIV, DNA methylation, methylation risk score, obesity

## Abstract

**Background/Objectives:** People with HIV (PWH) on antiretroviral therapy (ART) often gain weight, which increases their risk of type 2 diabetes and cardiovascular disease. The role of DNA methylation (DNAm) markers in obesity among PWH is understudied. This research explores the relationship between body mass index (BMI) and epigenetic patterns to better understand and manage obesity-related risks in PWH. **Methods:** We conducted an epigenome-wide association study (EWAS) on 892 African American male PWH from the Veterans Aging Cohort Study, examining BMI associations with DNAm using linear mixed models, adjusting for covariates, including soluble CD14. We compared our results with BMI-associated DNAm markers from non-HIV individuals and developed a methylation risk score (MRS) for BMI using machine learning and a cross-validation approach. **Results:** We identified four epigenome-wide significant CpG sites, including one in the *RAP1B* gene, indicating shared and unique BMI-related epigenetic markers between PWH and non-HIV individuals. The constructed BMI MRS explained approximately 19% of the BMI variance in PWH. **Conclusions:** DNAm markers and MRS are significantly linked to BMI in PWH, suggesting shared and distinct molecular mechanisms with non-HIV populations. These insights could lead to targeted interventions to reduce cardiometabolic disease risks in PWH under ART.

## 1. Introduction

As advances in treatment options for Human Immunodeficiency Virus (HIV) continue to expand, the number of people living with HIV (PWH) as a chronic condition is increasing. While the number of new infections has declined since its peak in 1995, those who were previously diagnosed are living longer [1]. As of 2022, more people are aging with HIV than ever before, an estimated 39 million globally. This aging population experiences comorbidities related to the virus and associated treatment regimens. Obesity and its sequelae are a growing concern in this population [2].

Prospective data from the United States Military HIV Natural History Study showed that the prevalence of obesity at the time of HIV diagnosis increased four-fold between 1985–1990 and 1996–2004 (3% to 12%) [3]. While antiretroviral therapy (ART) has significantly improved the prognosis for those with HIV, it has also been accompanied by complex changes in weight. A longitudinal study of ART-naïve patients found that 46% of the population was overweight or obese at baseline, increasing to 56% after 24 months [4]. However, this study showed only a modest effect of ART on BMI changes, suggesting other factors, such as immunologic recovery, may influence body weight dynamics in this context.

Obesity presents a higher risk of complications for PWH than for people without HIV (PWoH). Both HIV and obesity affect immunologic and inflammatory pathways in ways that increase the risk of negative outcomes [5]. Obesity is associated with developing pre-diabetes, type 2 diabetes (T2D), and cardiovascular disease (CVD) [6]. PWH who gain weight are more likely to develop diabetes than those gaining weight without HIV. Veteran Aging Cohort Study (VACS) data found that 48% of PWH gained 5 pounds or more after ART initiation, while only 31% of those without HIV gained the same amount of weight over one year, and for every 5 pounds gained, PWH had a 14% increased risk of developing T2D compared to 8% in PWoH during a 5-year follow-up [7]. These findings were found in other studies indicating a risk association between weight gain and T2D and CVD in PWH [5,8]. Specifically, for every body mass index (BMI) unit increase among those who had a normal weight at baseline, there is an 18–20% increased risk of CVD in 5 years [8]. These findings highlight the significant impact of weight management on PWH health outcomes, making BMI a critical marker for monitoring and potentially intervening in health risk trajectories.

Epigenome-wide association studies (EWASs) examine genome-wide epigenetic variants to identify potential associations with specific phenotypes, such as BMI [9,10]. The methylation of cytosine nucleotides at these sites is associated with changes in how DNA is transcribed, which in turn is associated with variations in gene expression levels that may contribute to or protect from illness [11]. Measured differences in methylation levels can be associated with the effects of certain exposures, such as aging or viral infection [11,12]. Earlier EWASs have investigated the role of obesity, cardiometabolic diseases, HIV, and ART on the epigenome, but none have been focused on ART-treated PWH [13,14,15,16,17,18,19].

Furthermore, a methylation risk score (MRS) can be developed to capture the combined association of multiple CpG sites with BMI [20]. The MRS is a weighted sum of methylation levels based on previously identified CpG sites associated with a trait. After proper training, testing, and validation, the MRS can aggregate individual epigenetic associations to explain a larger amount of phenotypic variance and can serve as a predictive tool for assessing the risk of obesity and its associated health outcomes. By identifying individuals at a higher genetic risk for an increased BMI, targeted interventions can be implemented to mitigate this risk, thereby enhancing patient care and potentially reducing the burden of obesity-related health issues. This study aimed to identify individual BMI-associated CpG sites and evaluate the MRS of BMI in a PWH cohort who self-reported as African American, a demographic that has been underrepresented in previous research and may exhibit distinct epigenetic patterns relevant to obesity and its associated health outcomes.

## 2. Results

The demographic and clinical characteristics in the 450 K and 850 K groups were similar, as shown in Table 1. Participants in the VACS-450 K (N = 466) and VACS-850 K (N = 426) cohorts were self-reported African American males and had an average age of 52.2 ± 7.5 and 50.8 ± 8.0 years, respectively. VACS-850 K exhibited a slightly higher average BMI of 26.2 ± 5.23 kg/m^2^, compared to 25.5 ± 4.7 kg/m^2^ in VACS-450 K (*p* = 0.040). The prevalence of hepatitis C virus infection was significantly higher in VACS-450 K (54.9%) compared to VACS-850 K (32.2%) (*p* < 0.0001).

### 2.1. EWAS of BMI

**Figure 1:**
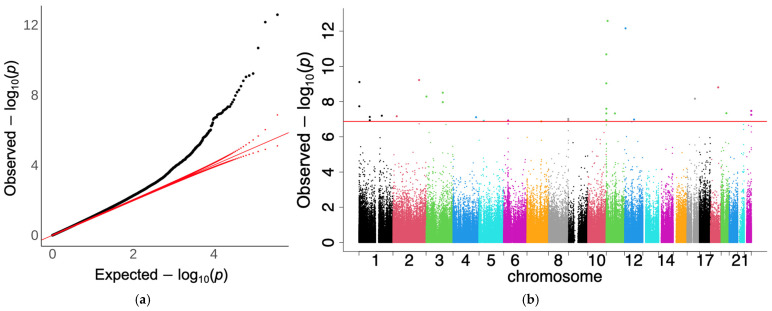
Quantile–quantile (QQ) plot and Manhattan plot for the meta-analysis of EWAS of the body mass index. Association models were adjusted for covariates: age, hepatitis C virus infection, diabetes status, smoking status, HIV viral load, and computed cell-type proportions. The Y-axis shows the −log10 (observed *p*-values) grouped by each chromosome in assorted colors (X-axis). (**a**) QQ-plot for the adjusted *p*-values using Bacon (inflation factor: 1.07). The red lines indicate the distribution of expected *p*-values (solid diagonal line) and their 95% confidence interval (dotted lines). (**b**) Manhattan plot for adjusted *p*-values using Bacon. Abbreviations: EWAS, epigenome-wide association study. Red lines indicate genome-wide significance after adjusting for multiple testing.

The initial BMI EWAS identified 84 significant CpG sites in VACS-450 K (Table S1, Figure S1) and 41 CpG sites in VACS-850 K (Table S2, Figure S2) with an FDR-q < 0.05. Using the Bonferroni-corrected p-value threshold, 23 and 13 CpG sites were associated with BMI in VACS-450 K and VACS-850 K, respectively. In the meta-analysis, 124 CpG sites were found to be significantly associated with BMI using an FDR-q < 0.05 (Table S3). Using the Bonferroni-corrected *p*-value threshold, 30 CpG sites remained significant (Table 2). The quantile-quantile (QQ) plot (Figure 1a) comparing observed to expected *p*-values in BMI meta-analyses revealed trivial global inflations (inflation factor (IF): 1.07). These CpG sites were mapped to the *IFITM1*, *STAT1*, *PRDM16*, *PARP9*, *IL5RA*, *SKI*, *IRF7*, *KLHDC7B*, *DPF1*, *CD247*, *IFT172*, *IFI44L*, *DDX60*, *RAP1B*, *PSMB8*, *PSMB8-AS1*, and *SRPK2* genes (Figure 1b). Notably, a significant proportion of the CpG sites from our meta-analysis overlapped with those identified in a previous sCD14 EWAS conducted on the same cohort [17]. To better understand the potential influence of sCD14 on the observed BMI associations, we incorporated sCD14 levels as an additional covariate within our EWAS model.

**Figure 2:**
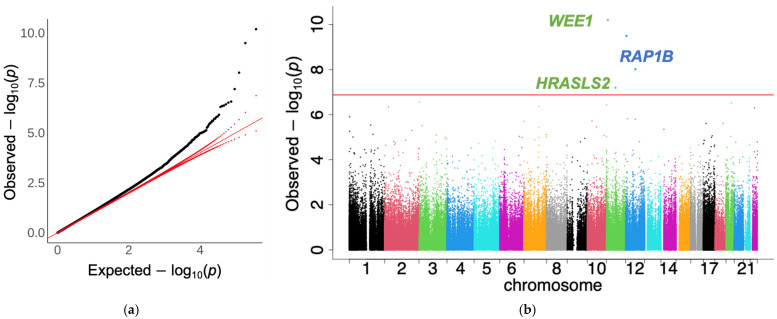
Quantile–quantile (QQ) plot and Manhattan plot for the meta-analysis of the EWAS of body mass index adjusting for sCD14. Association models were adjusted for covariates: sCD14 levels, age, hepatitis C virus infection, diabetes status, smoking status, HIV viral load, and computed cell-type proportions. (**a**) QQ-plot for the adjusted *p*-values using Bacon (inflation factor: 1.08). The red lines indicate the distribution of expected *p*-values (solid diagonal line) and their 95% confidence interval (dotted lines). (**b**) Manhattan plot for adjusted *p*-values using Bacon. Abbreviations: CpG, cytosine-phosphate-guanine dinucleotide; EWAS, epigenome-wide association study; sCD14, soluble cluster of differentiation 14. Red lines indicate genome-wide significance after adjusting for multiple testing. Gene annotations for significant CpG sites are shown.

Upon incorporating sCD14 levels as an additional covariate in the model, a notable reduction in the number of significant CpG sites was observed. The QQ and Manhattan plot results of the 450 K and 850 K EWAS of BMI, after adjusting for sCD14, are displayed in Figures S3 and S4. For the meta-analysis, only 15 CpG sites remained significant after multiple testing corrections with an FDR-q < 0.05. Using a more stringent Bonferroni-corrected *p*-value threshold, four CpG sites remained significant (cg17061862, cg10601624, cg04907505, and cg06178669). Among these, only cg04907505 was uniquely annotated to the neighboring region of the *RAP1B* gene (Table 3, Figure S5). The QQ plot (Figure 2a) comparing observed to expected *p*-values in BMI meta-analyses revealed trivial global inflations (IF: 1.08).

**Figure 3:**
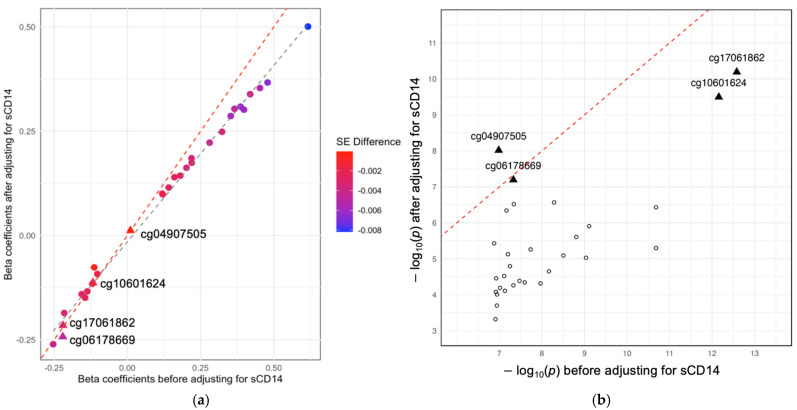
Comparison of effect sizes and *p*-values for BMI-associated CpG sites before and after adjusting for sCD14. (**a**) Each point’s color gradient, ranging from blue to red, represents the difference in the standard error (SE) before and after adjustment; blue indicates a smaller difference, while red indicates a larger difference. (**b**) The X-axis denotes the -log10 (expected *p*-values) before the sCD14 adjustment, and the Y-axis represents the −log10(observed *p*-values) after the sCD14 adjustment. Triangle markers denote the CpG sites that remained significant in the meta-analyses of the BMI EWAS adjusted for sCD14 after applying a Bonferroni-corrected *p*-value threshold (cg17061862, cg10601624, cg04907505, cg06178669). The red dashed line illustrates the diagonal line where the effect sizes are identified, and the grey dashed line illustrates the linear fit between the two sets of beta coefficients. Abbreviations: BMI, body mass index; CpG, cytosine-phosphate-guanine dinucleotide, sCD14, soluble cluster of differentiation 14.

To visualize the shift in effect sizes before and after adjusting for sCD14, a scatter plot was created comparing the effect sizes and p-values of the original 30 significant CpG sites from the meta-analysis of the BMI EWAS with their corresponding effect sizes after an adjustment (meta-analysis of BMI EWAS after adjusting for sCD14) (Figure 3, Table S5).

### 2.2. Comparison with BMI-Associated CpG Sites from Non-HIV Population

**Figure 4:**
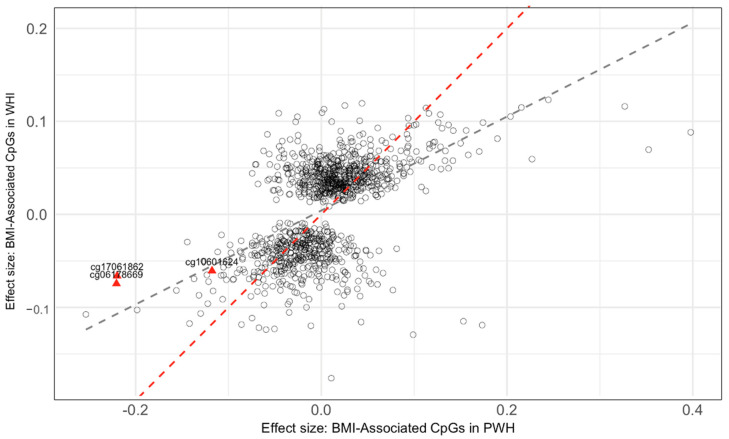
Comparison of effect sizes for BMI-associated CpG sites: BMI EWAS of people with HIV (PWH) vs. replicated CpG sites in Women’s Health Initiative (WHI). Triangle markers denote the CpG sites that remained significant in the meta-analyses of the BMI EWAS adjusted for sCD14 after applying a Bonferroni-corrected *p*-value threshold (cg17061862, cg10601624, cg06178669) and successfully replicated in WHI. A red dashed line illustrates the diagonal line where the effect sizes are identical, and a grey dashed line illustrates the linear fit between the two sets of effect sizes (beta-coefficients). Abbreviations: BMI, body mass index; CpG, cytosine-phosphate-guanine dinucleotide.

In our meta-analysis, we assessed the overlap of BMI-associated CpG sites with those identified in a previous study comprising 1238 significant BMI-associated CpG sites that were identified in the Women’s Health Initiative (WHI) cohort [21]. We found that 1063 of these CpG sites were common to both our meta-analysis results and the reported dataset. Among these 1063 shared CpG sites, 747 (70.3%) demonstrated directional consistency in their association with BMI (binomial test *p* < 2.2 × 10^−16^). A scatterplot of these shared CpG sites showed a modest positive correlation between effect sizes from the meta-analysis in PWH and those replicated in the WHI study [21], with R^2^ = 0.29, *p* < 0.001 (Figure 4). When we applied a Bonferroni correction for multiple comparisons with a threshold *p*-value of 0.05 divided by the number of common CpG sites (0.05/1063), 35 of these shared CpG sites remained statistically significant in our meta-analysis (Table S6).

### 2.3. Pathway Enrichment Analysis

**Figure 5:**
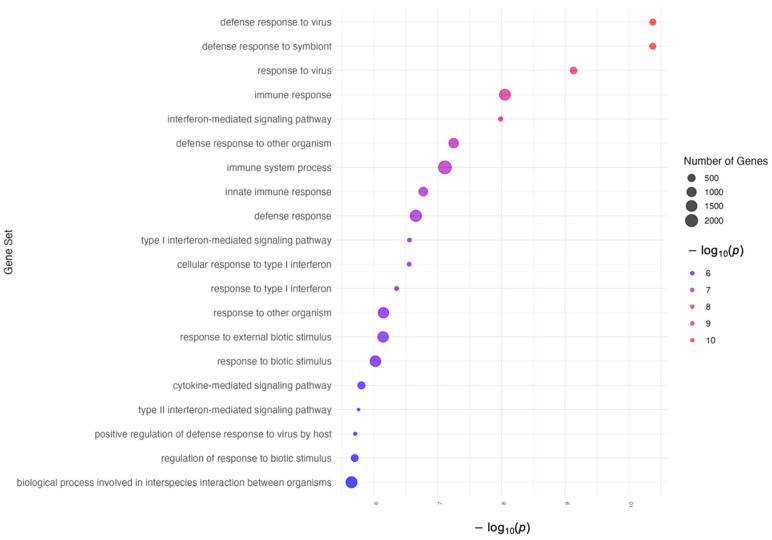
Enrichment analysis of top Gene Ontology terms.

The pathway enrichment analysis was conducted on the 30 CpG sites that were found to be Bonferroni-significant in the BMI EWAS meta-analysis, involving 18 unique genes. Gene Ontology (GO) analysis of these genes identified a network of enriched pathways predominantly related to immune responses and viral processes with a controlled false discovery rate (FDR-q < 0.05). The pathways and associated genes are extensively detailed in Table S7, with visual representations provided in Figure 5.

### 2.4. Methylation Risk Score

The direct MRS calculated by multiplying VACS DNA methylation beta values and the regression coefficients for 349 CpG sites from Do et al. [21] accounted for 18.9% of the variance in BMI among PWH from the VACS datasets (Figure S6). The internally developed Elastic-Net models based on 1079 CpG sites assessed via a 5-fold cross-validation process, demonstrated a median R-squared value ranging from 22.7% to 24.2% indicative of the model’s predictive accuracy (Table 4, Figures S7–S9).

## 3. Discussion

We employed an EWAS and MRS to examine the association between BMI and DNAm patterns among African American PWH. The EWAS identified four CpG sites significantly associated with BMI, after adjusting for relevant confounders, including sCD14, due to its potential influence on DNAm and BMI. Furthermore, we cross-referenced our CpG sites in the meta-analysis with those identified in a large EWAS meta-analysis of BMI and found that 70.3% of common CpG sites showed directional consistency among PWH (binomial test *p* < 2.2 × 10^−16^). Additionally, Elastic-Net regression models that incorporate factors, such as age, smoking, and VL, were constructed by applying machine learning algorithms to 349 previously reported BMI-associated CpG sites [21]. These models accounted for 18.9% of BMI variance among PWH, demonstrating the MRS’s substantive predictive utility for BMI in this population. This contrasts with the 32% variance reported in the non-HIV population, implying that potentially unique epigenetic mechanisms influence BMI among PWH [21].

In the meta-analysis, we identified four CpG sites significantly associated with BMI. Three of these sites (cg17061862, cg10601624, and cg06178669) demonstrated that hypomethylation (i.e., lower levels of methylation) is associated with higher BMI in the meta-analysis. Specifically, for each one-unit increase in BMI, we observed reductions in methylation of approximately 0.216%, 0.113%, and 0.243% at these sites, respectively. These findings align with prior research in PWoH, supporting the concept of an epigenetic mechanism that corresponds to changes in BMI independent of the HIV status. Further reinforcing their relevance, these three CpG sites have been associated with obesity-related traits in various studies [22,23,24,25]. Notably, cg17061862 exhibited a significant longitudinal association with BMI in a cohort consisting of 490 African American participants (beta = 0.001, *p*-value = 7.1 × 10^−6^) [22]. Likewise, cg10601624 showed a negative correlation with waist circumference (beta = −0.065, *p*-value = 2.1 × 10^−5^) in a study with multi-ethnic Asian individuals [26], and cg06178669 was linked to reduced methylation in association with obesity among 700 African American youths and young adults (beta = −0.005, *p*-value = 3.1 × 10^−3^) [27]. While these three CpG sites have been associated with obesity-related traits in various studies, it is important to note that they have not been mapped to any known genes, which suggests that their influence on BMI may be through regulatory mechanisms that are yet to be fully understood. In our comparative analysis with data from the WHI, which predominantly examined a cohort of White women, these three CpG sites were consistently identified, underscoring that such methylation changes are prevalent across various ethnic groups and sexes and are not confined to PWH [28].

In contrast, cg04907505, annotated to the *RAP1B* gene, exhibited a contrasting pattern of modest hypermethylation (i.e., higher levels of methylation), increasing by 0.012% per BMI unit increase in the meta-analysis [29,30]. The RAP1B gene, a member of the RAS superfamily of small GTP-binding proteins, plays a key role in cell adhesion, growth, and differentiation. One study reported the associations between hepatitis B virus-related hepatocellular carcinoma and the up-regulation of Rap1b, suggesting its involvement in cellular responses to viral infection [31]. The observed hypermethylation at this site may represent an epigenetic adaptation to HIV infection among PWH. This hypothesis is further supported by our Gene Ontology analysis, which shows a significant enrichment in immune and viral response pathways. Many of these pathways are interrelated and not mutually exclusive, reflecting the complex interplay between immune regulation and viral pathogenesis that may influence BMI. However, cg04907505’s methylation has not been previously linked to adiposity or related phenotypes and was also not identified in our comparative analysis. This could indicate unique metabolic complexities or a distinct role of the *RAP1B* gene in BMI regulation among PWH. The cg04907505 was also not found in the WHI dataset, which underscores the unique findings in our PWH cohort. This absence may indicate that our observed hypermethylation pattern may be a distinctive feature relevant to HIV-related, ancestry-related, or sex-related metabolic regulation. Further research is warranted to investigate whether this methylation site is implicated in the metabolic processes of the PWH population and to understand its potential role in the broader context of epigenetic modifications associated with HIV.

Adjusting for sCD14 levels led to a significant decrease in the number of BMI-associated CpG sites identified, suggesting a critical role for sCD14 in the epigenetic responses to BMI changes in PWH. In the general population, chronic inflammation and immune dysregulation are established consequences of obesity, often manifested as macrophage accumulation in adipose tissue and subsequent systemic inflammation, which in turn can precipitate metabolic disorders [32,33]. Notably, sCD14 serves as a soluble indicator of monocyte/macrophage activity, a process that is particularly pertinent in HIV due to the virus’s known impact on immune regulation [34,35]. Corroborating this, findings from a prior VACS study indicate that obese individuals with HIV exhibit lower levels of sCD14 compared to their non-obese counterparts [36]. Contrarily, other studies have reported elevated sCD14 levels in PWH irrespective of their weight status [37,38]. This discrepancy may be explained by considering the impact of steatohepatitis, a condition commonly associated with obesity and HIV, which could attenuate sCD14 production due to reduced hepatocyte activity [39]. Thus, these observations suggest that sCD14 not only reflects immune activation but may also serve as a critical modulator of the pathway linking BMI to epigenetic alterations, thereby underscoring its potential as a key factor in the epigenetic regulation associated with obesity in the context of HIV. Future research unraveling these connections could be crucial for developing interventions that target the synergistic impact of inflammation and obesity in PWH.

In addition, MRS computed from 349 CpG sites explained 18.9% of the variance in BMI among PWH. According to previous studies, the BMI variance explained by such scores in general populations ranges from 4.7% to 32% [21,40,41,42,43]. The unique metabolic alterations and the impact of ART on body composition inherent to PWH highlight the importance of our findings in this population. Our results from Elastic-Net models show that MRS within PWH explains 19% of the variability in BMI, which is higher than the best available genetic risk scores (~10%) [44,45]. The results indicate a potential for applying these findings to disease risk stratification that accounts for both genetic and environmental factors, enhancing our understanding of BMI determinants within this demographic. For individuals with HIV who are at heightened risk for obesity or related metabolic disorders, the application of MRS in clinical evaluations could lead to the early adoption of personalized lifestyle and dietary measures. Additionally, our insights offer the potential to tailor ART regimens more effectively and to consider the integration of novel pharmacological interventions, thereby enhancing strategies for weight management and improving overall metabolic health.

The strength of our study lies in its targeted focus on PWH of self-reported African Americans, a demographic that may exhibit distinct epigenetic patterns and ART responses and has historically been understudied in epigenetic research. This focus is critical as it sheds light on the distinct interactions among HIV, ART, and metabolic processes, which may differ in this demographic due to genetic and lifestyle factors. Furthermore, this research adds substantial public health importance considering the rising prevalence of obesity in PWH, which is known to increase the risk of developing co-morbid conditions.

This study has several limitations. First, the meta-analysis was limited to CpG sites common to both the 450 K and 850 K array platforms, potentially narrowing the epigenetic discovery scope. Additionally, while the assessment of DNA methylation in peripheral blood cells is informative for immune function analysis in HIV, it may not completely represent epigenetic associations in adipose and brain tissues, which are more directly involved with obesity. Lastly, the inclusion of only male participants also limits the generalizability of the results, indicating a need for subsequent studies to include women to validate these findings broadly.

## 4. Materials and Methods

### 4.1. Study Population

The VACS is a prospective study of Veterans receiving care at Department of Veterans Affairs medical sites across the United States [46]. This study utilized clinical, biomarker, and DNAm data from the VACS Biomarker Cohort, which is composed of whole blood samples taken between 2005 and 2007 from VACS participants with (N = 1525) and without (N = 843) HIV [47]. The current study utilizes DNA methylation data from PWH in the VACS-Biomarker Cohort. As this study is based on secondary analysis of de-identified data, no additional informed consent or Institutional Review Board (IRB) approval was required. The original VACS Biomarker Cohort study, which involved the collection of blood samples and data between 2005 and 2007, was approved by the Institutional Review Boards at all VACS locations, and written informed consent was obtained from all participants.

### 4.2. Data Collection

Clinical and demographic data closest to the date of blood sample collection were retrieved from electronic health records. These included age, sex, BMI, self-reported ancestry, chronic health conditions, medication use, plasma HIV-1 RNA viral load (VL), hepatitis B infection status, hepatitis C infection status, and cigarette smoking status. The exposure of interest was BMI, defined as weight in kilograms divided by height in meters squared (kg/m^2^). Hepatitis B and C infection statuses were both defined as positive or negative. Information on cigarette smoking status was gathered from VACS surveys.

### 4.3. Laboratory Procedures

Serum levels of the soluble cluster-of-differentiation-14 (sCD14) protein were quantified using an enzyme-linked immunosorbent assay (Quantikine sCD14 immunosorbent assay, R&D Systems, Minneapolis, MN, USA). The measurable range of sCD14 was 40–3200 ng/mL, utilizing a sample dilution of 200-fold and four control samples [48].

### 4.4. DNA Methylation and Quality Control

One subset of blood samples underwent epigenome-wide CpG sites profiling using the Illumina Infinium Human Methylation 450 K BeadChip (VACS-450 K) (Illumina, Inc., San Diego, CA, USA) [49], and another subset was tested using the newer generation of the DNA methylation array—Illumina Methylation EPIC (850 K) BeadChip (VACS-850 K) (Illumina, Inc., San Diego, CA, USA) [50]. Quality control measures, including data normalization and batch effect correction through control-probe adjustments, were standardized as previously detailed [16]. The minfi package in R [51] was used to quantile-normalize all raw intensity values for each probe. Normalized intensity data were then utilized to establish a methylation β value for each CpG site, ranging from 0 to 1, with 0 representing complete unmethylation and 1 representing complete methylation. Proportions of six blood cell types (CD4+ T cells, CD8+ T cells, natural killer T cells, B cells, monocytes, and granulocytes) were calculated using cell-type specific CpG sites from a reference panel implemented in the R minfi package [52]. After quality control, the VACS-450 K data set had 412,583 CpG sites, and the VACS-850 K data set contained 846,604 CpG sites, with a combined total of 374,357 unique CpG sites. The CpG sites measured using the 850 K and 450 K chips were mapped to Genome Research Consortium human build 37 (GRCh37)/hg19.

### 4.5. Statistical Model

The study was restricted to the analysis of 892 self-reported African American, non-Hispanic male PWH, and no genetic testing was conducted to ascertain ancestry. We compared the demographic and clinical characteristics of VACS450 K and VACS850 K using standard two-sample t-tests for numeric variables and chi-squared tests for categorical data. EWASs were conducted individually for the 450 K and 850 K arrays. In each of these, linear mixed-effect regression models were employed from the coMet R library (version 1.34.0) to evaluate the relationship between DNA methylation at individual CpG sites and BMI [53]. The exposure of interest was BMI (continuous), the outcome was blood DNA methylation, with the following covariates were adjusted: age (continuous), hepatitis C virus infection (binary), diabetes status (binary), smoking status (never, current, and previous), absolute VL (continuous), and computed cell-type proportions (percentage CD4+ T-cells, CD8+ T-cells, B cells, NK cells, monocytes, and granulocytes). To account for batch effects, chip ID was incorporated as a random effect. Further adjustments for p-values were made using the Bacon R package to adjust for potential unmeasured bias and cryptic relatedness [54], particularly for the 850 K array and the following meta-analysis. This approach was selected for the 850 K array due to its increased probe density and complexity, which may introduce greater variability and potential batch effects that are not as prevalent in the 450 K array. The Bacon method helps to correct for these factors, ensuring that the results are robust and reliable. Similarly, in the meta-analysis, where data from different cohorts are combined, this method helps to mitigate heterogeneity and potential confounding that could arise from pooling diverse datasets.

A meta-analysis was performed to integrate the findings from the 450 K and 850 K arrays. For this, METAL software (version released on 25 March 2011) was used, executing a fixed-effect model with inverse variance weighting [21]. Epigenome-wide significance was set at a Benjamini–Hochberg false discovery rate (FDR-q) of 0.05, and this threshold was applied to both the individual array analyses and the meta-analysis to maintain consistency in significance testing across all data sets. The Bonferroni level was also applied for statistical significance to allow for multiple testing (450 K: 0.05/412, 583 CpGs = 1.2 × 10^−7^, 850 K: 0.05/846, 604 CpGs = 6.0 × 10^−8^, meta-analysis: 0.05/374, 357 CpGs = 1.3 × 10^−7^). The regression beta coefficients, standard errors, and p-values from the meta-analysis of single-CpG analyses were included in the input files for these analyses. To evaluate the surrounding signals of the identified BMI-associated CpG sites, the coMet R package (version 1.34.0) was used to create regional plots [53]. These plots included all tested CpG sites within a 5000-base-pair radius of each significant association. Additionally, sCD14 levels were included as a covariate in our EWAS model due to their established role in immune function and their association with metabolic processes related to obesity. This inclusion is supported by the overlap of CpG sites associated with sCD14 in a prior EWAS conducted on the same cohort, suggesting a potential interaction among sCD14, DNA methylation, and BMI.17

### 4.6. Comparison with BMI-Associated CpG Sites from a Population Without HIV

Without a directly comparable BMI EWAS specifically on PWH, we compared our findings with a previously published BMI EWAS on PWoH [21]. The meta-analysis integrated all CpG sites analyzed in our study to identify overlaps with CpG sites that were genome-wide significant in the previous study [21]. This comparative analysis employed linear regression to assess the correlation of the effect sizes (beta coefficients) for CpG sites identified in our study and the previous study [21].

### 4.7. Pathway Enrichment Analysis

Gene Ontology (GO) analysis was conducted based on annotated genes using epigenome-wide significant CpG sites from our meta-analysis results. The analysis was completed using the missMethyl R package (version 1.36.0), which was corrected for the distribution of the CpG sites on the array. Thirty-three Significant pathways were defined as those with an FDR-q < 0.05.

### 4.8. Methylation Risk Score (MRS) of BMI

**Figure 6:**
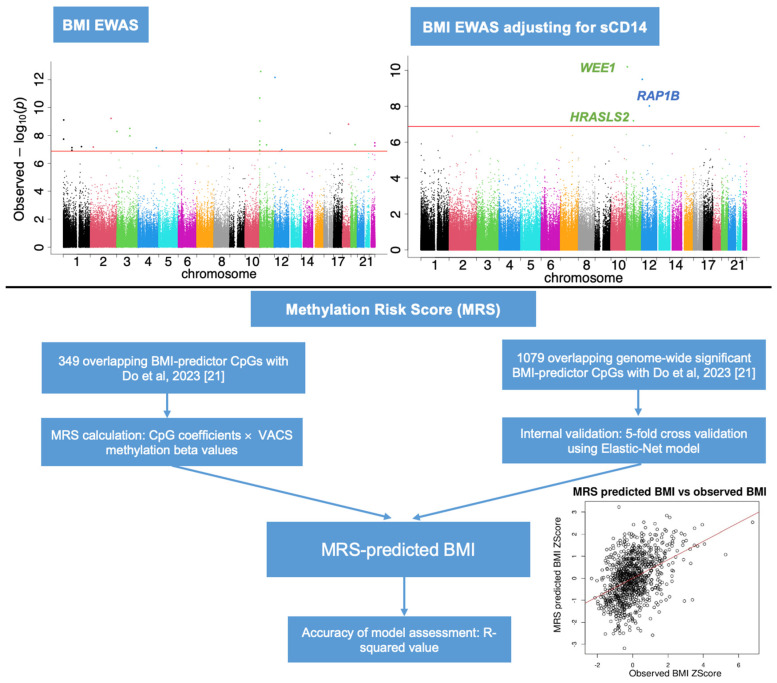
Workflow diagram for epigenome-wide and methylation risk score analysis of body mass index among people with HIV in the Veteran Aging Cohort Study (VACS). Abbreviations: BMI, body mass index; CpG, cytosine-phosphate-guanine dinucleotide; EWAS, epigenome-wide association study; sCD14, soluble CD14.

The EWAS conducted by Do et al. replicated 1238 CpG sites significantly associated with BMI among PWoH [21]. Using these sites, they developed an Elastic-Net model and identified 398 key CpG sites as predictors. In our study, we used these findings as a foundation to calculate an MRS for individuals in the VACS datasets. Out of the 398 CpG sites, 349 CpG sites were present in the VACS dataset. The regression coefficients of these 349 CpG sites derived from Do et al. were multiplied by their methylation beta values in the VACS dataset to calculate MRS. We further expanded on this work by constructing de-novo Elastic-Net models, as outlined in Figure 6. Specifically, within the VACS dataset, we applied Elastic-Net regression to the same 1238 genome-wide significant CpG sites highlighted by Do et al. [21]. Of these, 1079 were present and included in our analysis. We applied an Elastic-Net regression with a grid search method to determine the optimal alpha (α) and lambda (λ) values for our MRS of BMI. The grid search assessed combinations of α values ranging from 0 to 1 (in increments of 0.1) and 100 λ values for each α, using an internal 10-fold cross-validation within our dataset (N = 892). We controlled for potential confounders, including age (continuous), smoking status (never, current, and previous), absolute VL (continuous), and highly active antiretroviral therapy (HAART)(binary), during model training. Using the significant sites and coefficients selected by the model, the MRS was calculated by multiplying model-derived regression coefficients with the beta values of the CpG sites selected by the model. The performance of the BMI MRS model was assessed based on the median R-squared value obtained from 5-fold cross-validation, indicating the model’s predictive strength. Cross-validation was conducted using the cv.glmnet function from the R glmnet package (version 4.1.8) [55]. All statistical analyses were performed in the R statistical environment version 4.2.2 (http://www.r-project.org/).

## 5. Conclusions

In conclusion, this study found a significant association between BMI and DNAm markers and MRS among PWH. The consistency of the epigenetic associations between people with and without HIV shows that obesity-related epigenetic profiles share common biological pathways. Integrating these findings with EWAS of the general population, comprehensive epigenetic research on BMI in PWH could provide insights into obesity-related risk in the setting of HIV infection and ART therapy, potentially leading to further reductions in elevated chronic disease risks among PWH.

## Figures and Tables

**Table 1:** Population characteristics stratified by arrays in self-reported African Americans with HIV from the Veteran Aging Cohort Study.

Variable	450 K (N = 466)	850 K (N = 426)	*p*-Value
BMI (kg/m^2^), mean ± SD	25.5 ± 4.7	26.2 ± 5.23	0.040
Age (years), mean ± SD	52.2 ± 7.5	50.8 ± 8.0	0.007
sCD14 (ng/mL), mean ± SD	1838.0 ± 552.5	1781.6 ± 545.5	0.150
HIV Viral load (copies/mL), median (Q1, Q3)	75.0 (75.0, 418, 800.0)	75.0 (56.2, 709, 900.0)	0.706
CD4 counts, mean ± SD	412.1 ± 265.4	445.5 ± 287.4	0.073
Diabetes, *n* (%)	89 (19.1%)	79 (18.5%)	0.900
HCV infection, *n* (%)	256 (54.9%)	137 (32.2%)	<0.0001
Smoking Status			0.132
Nonsmoker, *n* (%)	92 (20.6%)	107 (25.1%)	
Current smoker, *n* (%)	277 (59.4%)	230 (54.0%)	
Past smoker, *n* (%)	97 (20.0%)	89 (20.9%)	

Abbreviations: BMI, body mass index; HCV, Hepatitis C virus infection; sCD14, soluble cluster of differentiation 14; SD, standard deviation.

**Table 2:** Meta-analyses of BMI-associated CpG sites in self-reported African Americans with HIV from the Veteran Aging Cohort Study.

CpG Sites	Chr	Position (bp)	Gene	Beta	SE	*p*-Value
cg17061862	11	9,590,431	NA	−0.2199	0.0301	2.63 × 10^−13^
cg10601624	12	6,404,377	NA	−0.1177	0.0164	6.95 × 10^−13^
cg23570810	11	315,102	*IFITM1*	0.4192	0.0626	2.08 × 10^−11^
cg14951497	2	191,875,807	*STAT1*	0.1805	0.0292	5.98 × 10^−10^
cg10493186	1	3,134,756	*PRDM16*	−0.2158	0.0351	7.77 × 10^−10^
cg03038262	11	315,262	*IFITM1*	0.3230	0.0528	9.22 × 10^−10^
cg15871086	18	56,526,595	NA	0.1194	0.0198	1.56 × 10^−9^
cg22930808	3	122,281,881	*PARP9*	0.6164	0.1040	3.13 × 10^−9^
cg23032421	3	3,152,038	*IL5RA*	−0.1371	0.0235	5.17 × 10^−9^
cg07839457	16	57,023,022	*NA*	0.4525	0.0781	6.86 × 10^−9^
cg08122652	3	122,281,939	*PARP9*	0.4786	0.0837	1.08 × 10^−9^
cg16704920	1	2,169,407	*SKI*	−0.1027	0.0183	1.85 × 10^−8^
cg08926253	11	614,761	*IRF7*	0.2807	0.0504	2.55 × 10^−8^
cg07833467	22	50,986,511	*KLHDC7B*	0.3528	0.0639	3.38 × 10^−8^
cg26950531	19	38,704,515	*DPF1*	−0.2536	0.0464	4.57 × 10^−8^
cg01971407	11	313,624	*IFITM1*	0.2196	0.0402	4.69 × 10^−8^
cg06178669	11	63,334,608	NA	−0.2206	0.0404	4.71 × 10^−8^
cg11829870	22	50,988,451	*KLHDC7B*	0.1607	0.0296	5.65 × 10^−8^
cg09554443	1	167,487,762	*CD247*	−0.1561	0.0289	6.34 × 10^−8^
cg23401251	2	27,683,905	*IFT172*	−0.1445	0.0268	6.81 × 10^−8^
cg13304609	1	79,085,162	*IFI44L*	0.3866	0.0718	7.42 × 10^−8^
cg05883128	4	169,239,131	*DDX60*	0.3656	0.0680	7.73 × 10^−8^
cg16400320	8	144,105,210	NA	0.1205	0.0226	9.66 × 10^−8^
cg04907505	12	69,004,265	*RAP1B*	0.0104	0.0020	1.04 × 10^−7^
cg09026253	11	313,267	*IFITM1*	0.2018	0.0381	1.14 × 10^−7^
cg03607951	1	79,085,586	*IFI44L*	0.3978	0.0750	1.15 × 10^−7^
cg08099136	6	32,811,251	*PSMB8*; *PSMB8-AS1*	0.2188	0.0413	1.19 × 10^−7^
cg12906975	8	144,105,259	NA	0.1413	0.0267	1.21 × 10^−7^
cg05478392	5	34,510,767	NA	−0.1134	0.0215	1.24 × 10^−7^
cg27395226	7	104,994,322	*SRPK2*	−0.1196	0.0227	1.33 × 10^−7^

Abbreviations: BMI, body mass index; bp, base-pair; Beta, beta-coefficient; Chr, chromosome; CpG, cytosine-phosphate-guanine dinucleotide; SE, standard error of beta coefficient. NA (not available) indicates that there are no gene annotations within a range of approximately 5000 base pairs from the significant association at this CpG site. Beta and SE were reported as coefficient × 100, which indicates the unit of a 1% methylation level increase. Linear mixed models were performed separately in VACS450 K and VACS850 K, followed by a meta-analysis for 374, 357 CpG sites common to VACS-450 K and VACS-850 K. Beta refers to the beta-coefficient estimates of the BMI change per 1% changes in the DNA methylation beta value, estimated through meta-analysis and linear mixed models in VACS 450 K and VACS 850 K, respectively. Base-pair positions of CpG sites were mapped to Genome Research Consortium human build 37 (GRCh37)/hg19.

**Table 3:** Meta-analyses of BMI-associated CpG sites Adjusted for sCD14 levels in self-reported African Americans with HIV from the Veteran Aging Cohort Study.

CpG Sites	Chr	Position (bp)	Gene	Beta	SE	*p*-Value
cg17061862	11	9,590,431	NA	−0.216	0.033	6.44 × 10^−11^
cg10601624	12	6,404,377	NA	−0.113	0.018	3.20 × 10^−10^
cg04907505	12	69,004,265	*RAP1B*	0.0112	0.002	9.68 × 10^−9^
cg06178669	11	63,334,608	NA	−0.243	0.045	6.41 × 10^−8^

Abbreviations: BMI, body mass index; bp, base-pair; Beta, beta-coefficient; Chr, chromosome; CpG sites, cytosine-phosphate-guanine dinucleotide sites; SE, standard error of beta coefficient. NA (not available) indicates that there are no gene annotations within a range of approximately 5000 base pairs from the significant association at this CpG site. Beta and SE were reported as the coefficient × 100, which indicates the unit of a 1% methylation level increase.

**Table 4:** The accuracy for the BMI predicted based on CpG sites in self-reported African Americans with HIV from the Veteran Aging Cohort Study.

Model: 1079 CpG Sites	Median Model R-Square
a. CpG only	24.2%
b. CpG + Age + Smoking	22.7%
c. CpG + Age + Smoking +VL + HAART	23.5%

Abbreviations: BMI, body mass index; CpG, cytosine-phosphate-guanine dinucleotide; HAART, highly active antiretroviral therapy; VL, viral load.

## Data Availability

Due to US Department of Veterans Affairs (VA) regulations and our ethics agreements, the analytic data sets used for this study are not permitted to leave the VA firewall without a Data Use Agreement. This limitation is consistent with other studies based on VA data. However, VA data are made freely available to researchers with an approved VA study protocol. For more information, please visit https://www.virec.research.va.gov (accessed on 2 December 2024) or contact the VA Information Resource Center at VIReC@va.gov.

## Supplementary Materials

The following supporting information can be downloaded at: https://www.mdpi.com/article/10.3390/epigenomes8040046/s1. Figure S1: 450 K EWAS for Body Mass Index; Figure S2: Results for the 850 K EWAS for Body Mass Index; Figure S3: Results for the 450 K EWAS for Body Mass Index after adjusting for sCD14; Figure S4: Results for the 850 K EWAS for Body Mass Index after adjusting for sCD14; Figure S5. Regional plots of significant CpG sites associated with body mass index from meta-analysis among African Americans in Veteran Aging Cohort Study; Figure S6. Methylation risk score for BMI calculated using 349 CpG regression coefficients and VACS DNA methylation beta values; Figure S7. Cross-Validation of Predictive Accuracy for BMI Variance Using Basic Elastic-Net Model on CpG Sites in PWH; Figure S8. Cross-Validation of Predictive Accuracy for BMI Variance Using Elastic-Net Models Incorporating Age and Smoking on CpG Sites in PWH; Figure S9. Cross-Validation of Predictive Accuracy for BMI Variance Using Elastic-Net Models Incorporating Age, Smoking, Viral Load, and Medication Data on CpG Sites in PWH. Table S1: BMI-Associated CpG Sites with FDR q < 0.05 in 450 K EWAS; Table S2. BMI-Associated CpG Sites with FDR q < 0.05 in 850 K EWAS; Table S3. BMI-Associated CpG Sites with FDR q < 0.05 in Meta-analysis EWAS; Table S4. BMI-Associated CpG Sites with FDR q < 0.05 in Meta-analysis EWAS after adjusting for sCD14; Table S5. Comparison of Effect Sizes and p-values Before and After Adjusting sCD14; Table S6. Comparative Analysis of BMI-Associated CpG Sites Between Meta-Analysis of EWAS and the WHI Cohort; Table S7. Gene Ontology Terms Enriched in Biological Process Categories with Statistical Significance.

## References

[1] Global HIV Statistics. https://www.unaids.org/sites/default/files/media_asset/UNAIDS_FactSheet_en.pdf.

[2] Henry T.A. Adult Obesity Rates Rise in 6 States, Exceed 35% in 7. https://www.ama-assn.org/delivering-care/public-health/adult-obesity-rates-rise-6-states-exceed-35-7.

[3] Crum-Cianflone N., Roediger M.P., Eberly L., Headd M., Marconi V., Ganesan A., Weintrob A., Barthel R.V., Fraser S., Agan B.K. (2010). Increasing rates of obesity among HIV-infected persons during the HIV epidemic. PLoS ONE.

[4] Tate T., Willig A.L., Willig J.H., Raper J.L., Moneyham L., Kempf M.C., Saag M.S., Mugavero M.J. (2012). HIV infection and obesity: Where did all the wasting go?. Antivir. Ther..

[5] McMahon C.N., Petoumenos K., Hesse K., Carr A., Cooper D.A., Samaras K. (2018). High rates of incident diabetes and prediabetes are evident in men with treated HIV followed for 11 years. AIDS.

[6] Savinelli S., Wrigley Kelly N.E., Feeney E.R., O’Shea D.B., Hogan A.E., Overton E.T., Landay A.L., Mallon P.W. (2022). Obesity in HIV infection: Host-pathogen interaction. AIDS.

[7] Herrin M., Tate J.P., Akgun K.M., Butt A.A., Crothers K., Freiberg M.S., Gibert C.L., Leaf D.A., Rimland D., Rodriguez-Barradas M.C. (2016). Weight Gain and Incident Diabetes Among HIV-Infected Veterans Initiating Antiretroviral Therapy Compared With Uninfected Individuals. J. Acquir. Immune Defic. Syndr..

[8] Achhra A.C., Mocroft A., Reiss P., Sabin C., Ryom L., de Wit S., Smith C.J., d’Arminio Monforte A., Phillips A., Weber R. (2016). Short-term weight gain after antiretroviral therapy initiation and subsequent risk of cardiovascular disease and diabetes: The D:A:D study. HIV Med..

[9] Campagna M.P., Xavier A., Lechner-Scott J., Maltby V., Scott R.J., Butzkueven H., Jokubaitis V.G., Lea R.A. (2021). Epigenome-wide association studies: Current knowledge, strategies and recommendations. Clin. Epigenet..

[10] Jang H.S., Shin W.J., Lee J.E., Do J.T. (2017). CpG and Non-CpG Methylation in Epigenetic Gene Regulation and Brain Function. Genes.

[11] Sun Y.V. (2014). The Influences of Genetic and Environmental Factors on Methylome-wide Association Studies for Human Diseases. Curr. Genet. Med. Rep..

[12] Salameh Y., Bejaoui Y., El Hajj N. (2020). DNA Methylation Biomarkers in Aging and Age-Related Diseases. Front. Genet..

[13] Nelson K.N., Hui Q., Rimland D., Xu K., Freiberg M.S., Justice A.C., Marconi V.C., Sun Y.V. (2017). Identification of HIV infection-related DNA methylation sites and advanced epigenetic aging in HIV-positive, treatment-naive U.S. veterans. AIDS.

[14] Mathur R., Hui Q., Huang Y., Gwinn M., So-Armah K., Freiberg M.S., Justice A.C., Xu K., Marconi V.C., Sun Y.V. (2019). DNA Methylation Markers of Type 2 Diabetes Mellitus Among Male Veterans with or Without Human Immunodeficiency Virus Infection. J. Infect. Dis..

[15] Titanji B.K., Gwinn M., Marconi V.C., Sun Y.V. (2022). Epigenome-wide epidemiologic studies of human immunodeficiency virus infection, treatment, and disease progression. Clin. Epigenet..

[16] Chen J., Huang Y., Hui Q., Mathur R., Gwinn M., So-Armah K., Freiberg M.S., Justice A.C., Xu K., Marconi V.C. (2020). Epigenetic Associations With Estimated Glomerular Filtration Rate Among Men With Human Immunodeficiency Virus Infection. Clin. Infect. Dis..

[17] Titanji B.K., Wang Z., Chen J., Hui Q., So-Armah K., Freiberg M., Justice A.C., Ke X., Marconi V.C., Sun Y.V. (2022). Soluble CD14-associated DNA methylation sites predict mortality among men with HIV infection. AIDS.

[18] Titanji B.K., Lee M., Wang Z., Chen J., Hui Q., Lo Re Iii V., So-Armah K., Justice A.C., Xu K., Freiberg M. (2022). Epigenome-wide association study of biomarkers of liver function identifies albumin-associated DNA methylation sites among male veterans with HIV. Front. Genet..

[19] Taylor J.Y., Huang Y., Zhao W., Wright M.L., Wang Z., Hui Q., Potts-Thompson S., Barcelona V., Prescott L., Yao Y. (2023). Epigenome-wide association study of BMI in Black populations from InterGEN and GENOA. Obesity.

[20] Chen J., Gatev E., Everson T., Conneely K.N., Koen N., Epstein M.P., Kobor M.S., Zar H.J., Stein D.J., Huls A. (2023). Pruning and thresholding approach for methylation risk scores in multi-ancestry populations. Epigenetics.

[21] Do W.L., Sun D., Meeks K., Dugue P.A., Demerath E., Guan W., Li S., Chen W., Milne R., Adeyemo A. (2023). Epigenome-wide meta-analysis of BMI in nine cohorts: Examining the utility of epigenetically predicted BMI. Am. J. Hum. Genet..

[22] Sun D., Zhang T., Su S., Hao G., Chen T., Li Q.Z., Bazzano L., He J., Wang X., Li S. (2019). Body Mass Index Drives Changes in DNA Methylation: A Longitudinal Study. Circ. Res..

[23] Vehmeijer F.O.L., Kupers L.K., Sharp G.C., Salas L.A., Lent S., Jima D.D., Tindula G., Reese S., Qi C., Gruzieva O. (2020). DNA methylation and body mass index from birth to adolescence: Meta-analyses of epigenome-wide association studies. Genome Med..

[24] Geurts Y.M., Dugue P.A., Joo J.E., Makalic E., Jung C.H., Guan W., Nguyen S., Grove M.L., Wong E.M., Hodge A.M. (2018). Novel associations between blood DNA methylation and body mass index in middle-aged and older adults. Int. J. Obes..

[25] Kupers L.K., Monnereau C., Sharp G.C., Yousefi P., Salas L.A., Ghantous A., Page C.M., Reese S.E., Wilcox A.J., Czamara D. (2019). Meta-analysis of epigenome-wide association studies in neonates reveals widespread differential DNA methylation associated with birthweight. Nat. Commun..

[26] Chen Y., Kassam I., Lau S.H., Kooner J.S., Wilson R., Peters A., Winkelmann J., Chambers J.C., Chow V.T., Khor C.C. (2021). Impact of BMI and waist circumference on epigenome-wide DNA methylation and identification of epigenetic biomarkers in blood: An EWAS in multi-ethnic Asian individuals. Clin. Epigenet..

[27] Wang X., Pan Y., Zhu H., Hao G., Huang Y., Barnes V., Shi H., Snieder H., Pankow J., North K. (2018). An epigenome-wide study of obesity in African American youth and young adults: Novel findings, replication in neutrophils, and relationship with gene expression. Clin. Epigenet..

[28] The Women’s Health Initiative Study Group (1998). Design of the Women’s Health Initiative clinical trial and observational study. Control. Clin. Trials.

[29] Chrzanowska-Wodnicka M. (2010). Regulation of angiogenesis by a small GTPase Rap1. Vasc. Pharmacol..

[30] Zhang L., Cui M., Song L., Zhang M., Zhang J. (2019). Function, Significance, and Regulation of Rap1b in Malignancy. Crit. Rev. Eukaryot. Gene Expr..

[31] Sheng Y., Ding S., Chen K., Chen J., Wang S., Zou C., Zhang J., Cao Y., Huang A., Tang H. (2014). Functional analysis of miR-101-3p and Rap1b involved in hepatitis B virus-related hepatocellular carcinoma pathogenesis. Biochem. Cell Biol..

[32] Mulder R.H., Neumann A., Cecil C.A.M., Walton E., Houtepen L.C., Simpkin A.J., Rijlaarsdam J., Heijmans B.T., Gaunt T.R., Felix J.F. (2021). Epigenome-wide change and variation in DNA methylation in childhood: Trajectories from birth to late adolescence. Hum. Mol. Genet..

[33] Khanna D., Khanna S., Khanna P., Kahar P., Patel B.M. (2022). Obesity: A Chronic Low-Grade Inflammation and Its Markers. Cureus.

[34] Leveque M., Simonin-Le Jeune K., Jouneau S., Moulis S., Desrues B., Belleguic C., Brinchault G., Le Trionnaire S., Gangneux J.P., Dimanche-Boitrel M.T. (2017). Soluble CD14 acts as a DAMP in human macrophages: Origin and involvement in inflammatory cytokine/chemokine production. FASEB J..

[35] Sanjurjo L., Castelblanco E., Julve J., Villalmanzo N., Tellez E., Ramirez-Morros A., Alonso N., Mauricio D., Sarrias M.R. (2023). Contribution of Elevated Glucose and Oxidized LDL to Macrophage Inflammation: A Role for PRAS40/Akt-Dependent Shedding of Soluble CD14. Antioxidants.

[36] Taylor B.S., So-Armah K., Tate J.P., Marconi V.C., Koethe J.R., Bedimo R.J., Butt A.A., Gibert C.L., Goetz M.B., Rodriguez-Barradas M.C. (2017). HIV and Obesity Comorbidity Increase Interleukin 6 but Not Soluble CD14 or D-Dimer. J. Acquir. Immune Defic. Syndr..

[37] Cassol E., Malfeld S., Mahasha P., van der Merwe S., Cassol S., Seebregts C., Alfano M., Poli G., Rossouw T. (2010). Persistent microbial translocation and immune activation in HIV-1-infected South Africans receiving combination antiretroviral therapy. J. Infect. Dis..

[38] Sattler F.R., He J., Letendre S., Wilson C., Sanders C., Heaton R., Ellis R., Franklin D., Aldrovandi G., Marra C.M. (2015). Abdominal obesity contributes to neurocognitive impairment in HIV-infected patients with increased inflammation and immune activation. J. Acquir. Immune Defic. Syndr..

[39] Zhou Z., Xu M.J., Gao B. (2016). Hepatocytes: A key cell type for innate immunity. Cell. Mol. Immunol..

[40] Shah S., Bonder M.J., Marioni R.E., Zhu Z., McRae A.F., Zhernakova A., Harris S.E., Liewald D., Henders A.K., Mendelson M.M. (2015). Improving Phenotypic Prediction by Combining Genetic and Epigenetic Associations. Am. J. Hum. Genet..

[41] Mendelson M.M., Marioni R.E., Joehanes R., Liu C., Hedman A.K., Aslibekyan S., Demerath E.W., Guan W., Zhi D., Yao C. (2017). Association of Body Mass Index with DNA Methylation and Gene Expression in Blood Cells and Relations to Cardiometabolic Disease: A Mendelian Randomization Approach. PLoS Med..

[42] McCartney D.L., Hillary R.F., Stevenson A.J., Ritchie S.J., Walker R.M., Zhang Q., Morris S.W., Bermingham M.L., Campbell A., Murray A.D. (2018). Epigenetic prediction of complex traits and death. Genome Biol..

[43] Reed Z.E., Suderman M.J., Relton C.L., Davis O.S.P., Hemani G. (2020). The association of DNA methylation with body mass index: Distinguishing between predictors and biomarkers. Clin. Epigenet..

[44] Khera A.V., Chaffin M., Wade K.H., Zahid S., Brancale J., Xia R., Distefano M., Senol-Cosar O., Haas M.E., Bick A. (2019). Polygenic Prediction of Weight and Obesity Trajectories from Birth to Adulthood. Cell.

[45] Shim I., Kuwahara H., Chen N., Hashem M.O., AlAbdi L., Abouelhoda M., Won H.H., Natarajan P., Ellinor P.T., Khera A.V. (2023). Clinical utility of polygenic scores for cardiometabolic disease in Arabs. Nat. Commun..

[46] Justice A.C., Modur S.P., Tate J.P., Althoff K.N., Jacobson L.P., Gebo K.A., Kitahata M.M., Horberg M.A., Brooks J.T., Buchacz K. (2013). Predictive accuracy of the Veterans Aging Cohort Study index for mortality with HIV infection: A North American cross cohort analysis. J. Acquir. Immune Defic. Syndr..

[47] Armah K.A., McGinnis K., Baker J., Gibert C., Butt A.A., Bryant K.J., Goetz M., Tracy R., Oursler K.K., Rimland D. (2012). HIV status, burden of comorbid disease, and biomarkers of inflammation, altered coagulation, and monocyte activation. Clin. Infect. Dis..

[48] Justice A.C., Freiberg M.S., Tracy R., Kuller L., Tate J.P., Goetz M.B., Fiellin D.A., Vanasse G.J., Butt A.A., Rodriguez-Barradas M.C. (2012). Does an index composed of clinical data reflect effects of inflammation, coagulation, and monocyte activation on mortality among those aging with HIV?. Clin. Infect. Dis..

[49] Zhang X., Justice A.C., Hu Y., Wang Z., Zhao H., Wang G., Johnson E.O., Emu B., Sutton R.E., Krystal J.H. (2016). Epigenome-wide differential DNA methylation between HIV-infected and uninfected individuals. Epigenetics.

[50] Shu C., Justice A.C., Zhang X., Marconi V.C., Hancock D.B., Johnson E.O., Xu K. (2021). DNA methylation biomarker selected by an ensemble machine learning approach predicts mortality risk in an HIV-positive veteran population. Epigenetics.

[51] Aryee M.J., Jaffe A.E., Corrada-Bravo H., Ladd-Acosta C., Feinberg A.P., Hansen K.D., Irizarry R.A. (2014). Minfi: A flexible and comprehensive Bioconductor package for the analysis of Infinium DNA methylation microarrays. Bioinformatics.

[52] Houseman E.A., Kelsey K.T., Wiencke J.K., Marsit C.J. (2015). Cell-composition effects in the analysis of DNA methylation array data: A mathematical perspective. BMC Bioinform..

[53] Martin T.C., Yet I., Tsai P.C., Bell J.T. (2015). coMET: Visualisation of regional epigenome-wide association scan results and DNA co-methylation patterns. BMC Bioinform..

[54] van Iterson M., van Zwet E.W., Consortium B., Heijmans B.T. (2017). Controlling bias and inflation in epigenome- and transcriptome-wide association studies using the empirical null distribution. Genome Biol..

[55] Friedman J., Hastie T., Tibshirani R. (2010). Regularization Paths for Generalized Linear Models via Coordinate Descent. J. Stat. Softw..

