# Supplementary material for: Epigenome-Wide and Methylation Risk Score Analysis of Body Mass Index Among People with HIV
Source: Epigenomes. 2024 Dec 12;8(4):46. doi: 10.3390/epigenomes8040046 (PMC11675887; doi:10.3390/epigenomes8040046)

SUPPLEMENTARY TABLES AND FIGURES

Table S1. BMI-Associated CpG Sites with FDR q < 0.05 in 450K EWAS.

CpG sites	Chr	Position (bp)	Gene	Beta-coefficient	SE	p-value	bonf_P	FDR-q
cg23570810	11	315102	*IFITM1*	0.0061	0.0009	1.04E-10	4.31E-05	4.31E-05
cg03038262	11	315262	*IFITM1*	0.0052	0.0008	2.37E-10	9.79E-05	4.90E-05
cg14951497	2	191875807	*STAT1*	0.0023	0.0004	2.25E-09	9.30E-04	2.52E-04
cg23025459	1	3134420	*PRDM16*	-0.0036	0.0006	2.44E-09	1.01E-03	2.52E-04
cg06872964	1	79085250	*IFI44L*	0.0063	0.0011	5.77E-09	2.38E-03	4.76E-04
cg10493186	1	3134756	*PRDM16*	-0.0035	0.0006	9.25E-09	3.81E-03	6.36E-04
cg14864167	8	66751182	*PDE7A*	0.0078	0.0013	1.41E-08	5.83E-03	6.85E-04
cg05696877	1	79088769	*IFI44L*	0.0089	0.0015	1.49E-08	6.15E-03	6.85E-04
cg10601624	12	6404377	*NA*	-0.0013	0.0002	1.49E-08	6.17E-03	6.85E-04
cg05883128	4	169239131	*DDX60*	0.0053	0.0009	2.36E-08	9.72E-03	9.61E-04
cg01971407	11	313624	*IFITM1*	0.0036	0.0006	2.56E-08	1.06E-02	9.61E-04
cg21549285	21	42799141	*MX1*	0.0099	0.0018	3.58E-08	1.48E-02	1.23E-03
cg22930808	3	122281881	*PARP9*	0.0086	0.0015	4.41E-08	1.82E-02	1.40E-03
cg08122652	3	122281939	*PARP9*	0.0066	0.0012	5.42E-08	2.23E-02	1.50E-03
cg22862003	21	42797588	*MX1*	0.0065	0.0012	5.90E-08	2.43E-02	1.50E-03
cg17061862	11	9590431	*NA*	-0.0021	0.0004	6.12E-08	2.53E-02	1.50E-03
cg11694510	11	313354	*IFITM1*	0.0021	0.0004	6.18E-08	2.55E-02	1.50E-03
cg12461141	11	5710654	*TRIM22*	0.0028	0.0005	6.58E-08	2.72E-02	1.51E-03
cg03607951	1	79085586	*IFI44L*	0.0062	0.0011	7.14E-08	2.95E-02	1.52E-03
cg22910549	22	50985117	*KLHDC7B*	0.0037	0.0007	7.37E-08	3.04E-02	1.52E-03
cg07833467	22	50986511	*KLHDC7B*	0.0056	0.0010	8.22E-08	3.39E-02	1.61E-03
cg07839457	16	57023022	*NLRC5*	0.0065	0.0012	9.56E-08	3.94E-02	1.79E-03
cg18533225	22	50986813	*KLHDC7B*	0.0041	0.0007	1.10E-07	4.55E-02	1.98E-03
cg00676801	2	191876673	*STAT1*	0.0020	0.0004	1.26E-07	5.20E-02	2.11E-03
cg06981309	3	146260954	*PLSCR1*	0.0055	0.0010	1.28E-07	5.27E-02	2.11E-03

cg05095590	7	2139259	*MAD1L1*	0.0033	0.0006	1.51E-07	6.25E-02	2.40E-03
cg08926253	11	614761	*IRF7*	0.0037	0.0007	1.86E-07	7.66E-02	2.84E-03
cg03642472	6	145670687	*NA*	-0.0011	0.0002	2.04E-07	8.41E-02	3.00E-03
cg09026253	11	313267	*IFITM1*	0.0029	0.0006	2.16E-07	8.89E-02	3.07E-03
cg08818207	6	32820355	*TAP1*	0.0040	0.0008	2.74E-07	1.13E-01	3.77E-03
cg10552523	11	313478	*IFITM1*	0.0040	0.0008	3.30E-07	1.36E-01	4.40E-03
cg13304609	1	79085162	*IFI44L*	0.0051	0.0010	3.65E-07	1.51E-01	4.55E-03
cg08924203	21	42798747	*MX1*	0.0032	0.0006	3.85E-07	1.59E-01	4.55E-03
cg04492263	13	113409145	*ATP11A*	-0.0014	0.0003	3.85E-07	1.59E-01	4.55E-03
cg11702942	8	144102584	*LY6E*	0.0024	0.0005	3.86E-07	1.59E-01	4.55E-03
cg16400320	8	144105210	*NA*	0.0015	0.0003	4.80E-07	1.98E-01	5.25E-03
cg15871086	18	56526595	*NA*	0.0015	0.0003	4.84E-07	1.99E-01	5.25E-03
cg03848588	9	32525008	*DDX58*	0.0015	0.0003	4.84E-07	2.00E-01	5.25E-03
cg06188083	10	91093005	*IFIT3*	0.0057	0.0011	5.06E-07	2.09E-01	5.35E-03
cg05552874	10	91153143	*IFIT1*	0.0050	0.0010	6.23E-07	2.57E-01	6.43E-03
cg16411857	16	57023191	*NLRC5*	0.0026	0.0005	6.87E-07	2.83E-01	6.91E-03
cg03587597	8	144105055	*NA*	0.0014	0.0003	7.52E-07	3.10E-01	7.39E-03
cg10521023	1	245411583	*KIF26B*	-0.0010	0.0002	8.27E-07	3.41E-01	7.93E-03
cg11927233	5	170816542	*NPM1*	0.0032	0.0006	9.44E-07	3.90E-01	8.85E-03
cg27395226	7	104994322	*SRPK2*	-0.0014	0.0003	1.11E-06	4.58E-01	1.01E-02
cg01190666	20	62204908	*PRIC285*	0.0025	0.0005	1.12E-06	4.63E-01	1.01E-02
cg12906975	8	144105259	*NA*	0.0017	0.0003	1.26E-06	5.18E-01	1.09E-02
cg18434560	11	317767	*NA*	0.0015	0.0003	1.26E-06	5.22E-01	1.09E-02
cg17394304	3	187453973	*BCL6*	-0.0008	0.0002	1.32E-06	5.44E-01	1.11E-02
cg08159663	16	57022486	*NLRC5*	0.0023	0.0005	1.37E-06	5.67E-01	1.13E-02
cg27222803	3	193310868	*OPA1*	0.0005	0.0001	2.06E-06	8.49E-01	1.65E-02
cg16672203	6	31744391	*C6orf27*	-0.0014	0.0003	2.08E-06	8.60E-01	1.65E-02
cg19974879	11	611692	*PHRF1*	0.0006	0.0001	2.15E-06	8.87E-01	1.65E-02
cg04927537	17	76976091	*LGALS3BP*	0.0038	0.0008	2.16E-06	8.92E-01	1.65E-02

cg24200753	3	19055533	*NA*	-0.0023	0.0005	2.41E-06	9.94E-01	1.78E-02
cg14293575	22	18635460	*USP18*	0.0047	0.0010	2.45E-06	1.00E+00	1.78E-02
cg01309328	6	32811253	*PSMB8*	0.0026	0.0005	2.45E-06	1.00E+00	1.78E-02
cg03546163	6	35654363	*FKBP5*	0.0036	0.0008	2.54E-06	1.00E+00	1.81E-02
cg05432003	11	312518	*IFITM1*	0.0030	0.0006	2.59E-06	1.00E+00	1.81E-02
cg13130398	1	174844397	*RABGAP1L*	0.0021	0.0004	3.04E-06	1.00E+00	2.09E-02
cg20098015	22	50971140	*ODF3B*	0.0046	0.0010	3.22E-06	1.00E+00	2.18E-02
cg22016995	11	614787	*IRF7*	0.0033	0.0007	3.44E-06	1.00E+00	2.29E-02
cg02556393	3	168866705	*MECOM*	0.0027	0.0006	3.56E-06	1.00E+00	2.33E-02
cg16644494	22	50971601	*ODF3B*	0.0018	0.0004	3.75E-06	1.00E+00	2.42E-02
cg19413066	17	76965347	*NA*	-0.0016	0.0003	4.08E-06	1.00E+00	2.59E-02
cg09363892	1	954619	*AGRN*	-0.0023	0.0005	4.62E-06	1.00E+00	2.87E-02
cg15350780	18	9105577	*NDUFV2*	0.0021	0.0005	4.67E-06	1.00E+00	2.87E-02
cg01079652	1	79118191	*IFI44*	0.0057	0.0012	5.28E-06	1.00E+00	3.21E-02
cg18187029	12	96650848	*ELK3*	-0.0011	0.0002	5.85E-06	1.00E+00	3.43E-02
cg11224765	22	50971109	*ODF3B*	0.0033	0.0007	5.87E-06	1.00E+00	3.43E-02
cg24304309	1	154577895	*ADAR*	0.0017	0.0004	5.90E-06	1.00E+00	3.43E-02
cg01028142	2	7004578	*CMPK2*	0.0045	0.0010	6.08E-06	1.00E+00	3.43E-02
cg21490635	12	13349273	*EMP1*	-0.0009	0.0002	6.11E-06	1.00E+00	3.43E-02
cg23771366	11	86510998	*PRSS23*	0.0020	0.0004	6.16E-06	1.00E+00	3.43E-02
cg15246895	17	17672475	*RAI1*	-0.0010	0.0002	6.91E-06	1.00E+00	3.80E-02
cg22642495	19	10197856	*C19orf66*	0.0024	0.0005	7.50E-06	1.00E+00	4.07E-02
cg17156491	2	43259508	*NA*	-0.0023	0.0005	8.18E-06	1.00E+00	4.38E-02
cg01681525	17	79374741	*BAHCC1*	0.0021	0.0005	9.09E-06	1.00E+00	4.79E-02
cg25325512	6	37142220	*PIM1*	0.0022	0.0005	9.32E-06	1.00E+00	4.79E-02
cg26651978	17	76965407	*NA*	-0.0013	0.0003	9.59E-06	1.00E+00	4.79E-02
cg08099136	6	32811251	*PSMB8*	0.0027	0.0006	9.69E-06	1.00E+00	4.79E-02
cg10482356	16	56328421	*GNAO1*	-0.0012	0.0003	9.71E-06	1.00E+00	4.79E-02
cg18396675	17	56605391	*4-Sep*	-0.0009	0.0002	9.73E-06	1.00E+00	4.79E-02

cg09762515	7	101556588	*CUX1*	-0.0015	0.0003	9.74E-06	1.00E+00	4.79E-02

* Abbreviations: BMI (body mass index), CpG site (cytosine-phosphate-guanine dinucleotide sites), Chr (Chromosome), bp (base-pair), bonf_p: Bonferroni-adjusted P-value, fdr_q: False Discovery Rate-adjusted Q-value, NA (not available), SE (Standard error), EWAS (Epigenome-wide association study)

Table S2. BMI-Associated CpG Sites with FDR q < 0.05 in 850K EWAS

CpG sites	Chr	Position (bp)	Gene	Beta-coefficient	SE	p-value	bonf_P	FDR-q
cg13559225	1	182758953	*NPL*	0.0022	0.0003	2.50E-12	1.99E-06	1.99E-06
cg11606286	15	53364073	NA	-0.0025	0.0004	1.32E-10	1.05E-04	4.68E-05
cg07608848	2	1647185	*PXDN*	-0.0017	0.0003	2.33E-10	1.86E-04	4.68E-05
cg00878069	10	1102168	*WDR37*	0.0004	0.0001	2.53E-10	2.02E-04	4.68E-05
cg22413215	16	3649876	*SLX4*	-0.0011	0.0002	2.93E-10	2.34E-04	4.68E-05
cg19681027	11	67888477	*CHKA*	0.0015	0.0002	7.35E-10	5.86E-04	9.32E-05
cg23858565	16	67595703	*CTCF*	0.0017	0.0003	8.99E-10	7.17E-04	9.32E-05
cg07884352	7	143152	NA	-0.0016	0.0003	9.35E-10	7.45E-04	9.32E-05
cg18957484	12	99038577	*APAF1;IKBIP*	0.0004	0.0001	1.78E-09	1.42E-03	1.58E-04
cg04247530	8	144997355	*PLEC*	-0.0015	0.0003	8.56E-09	6.83E-03	6.82E-04
cg17166282	13	103249241	*TPP2*	0.0002	0.0000	1.23E-08	9.77E-03	8.88E-04
cg16265113	11	120000607	*TRIM29*	-0.0023	0.0004	1.34E-08	1.07E-02	8.90E-04
cg01021437	9	40792159	*ZNF658*	0.0005	0.0001	5.39E-08	4.30E-02	3.31E-03
cg03387111	3	101292956	*PCNP*	0.0003	0.0001	6.71E-08	5.35E-02	3.82E-03
cg21684374	5	61567810	NA	-0.0012	0.0002	8.39E-08	6.69E-02	4.46E-03
cg21932484	7	98806168	*KPNA7*	-0.0013	0.0002	2.46E-07	1.96E-01	1.22E-02
cg07276831	11	92264986	*FAT3*	-0.0012	0.0002	2.84E-07	2.27E-01	1.27E-02
cg13859208	12	130649720	*FZD10*	-0.0026	0.0005	2.99E-07	2.38E-01	1.27E-02
cg13832201	17	19282521	*MAPK7;B9D1*	0.0020	0.0004	3.05E-07	2.43E-01	1.27E-02
cg23546343	14	101347284	*MIR433;RTL1*	-0.0017	0.0003	3.18E-07	2.54E-01	1.27E-02
cg06516096	14	55904696	*TBPL2*	-0.0044	0.0009	3.41E-07	2.72E-01	1.30E-02
cg11343124	8	103964262	*AZIN1-AS1*	-0.0012	0.0002	5.27E-07	4.20E-01	1.87E-02

cg14679202	22	41600978	*L3MBTL2*	-0.0025	0.0005	5.38E-07	4.29E-01	1.87E-02
cg13054613	3	7742036	*GRM7*	-0.0023	0.0005	6.77E-07	5.40E-01	2.25E-02
cg22043875	8	61653379	*CHD7*	-0.0020	0.0004	1.21E-06	9.64E-01	3.73E-02
cg03420040	16	1027383	*LMF1*	0.0022	0.0004	1.22E-06	9.69E-01	3.73E-02
cg23961229	10	45475292	*C10orf10;RASSF4*	-0.0012	0.0002	1.31E-06	1.00E+00	3.88E-02
cg27410202	19	14104641	*RFX1*	-0.0012	0.0002	1.39E-06	1.00E+00	3.95E-02
cg17061862	11	9590431	NA	-0.0024	0.0005	1.48E-06	1.00E+00	3.95E-02
cg02556924	15	101661693	NA	0.0022	0.0005	1.49E-06	1.00E+00	3.95E-02
cg02837935	16	1027604	*LMF1*	0.0025	0.0005	1.59E-06	1.00E+00	4.03E-02
cg16704920	1	2169407	*SKI*	-0.0014	0.0003	1.62E-06	1.00E+00	4.03E-02
cg13816136	12	388975	NA	-0.0009	0.0002	1.74E-06	1.00E+00	4.20E-02
cg26267764	17	4846173	*RNF167*	-0.0011	0.0002	1.86E-06	1.00E+00	4.36E-02
cg17658885	10	38299503	*ZNF33A*	0.0003	0.0001	1.96E-06	1.00E+00	4.36E-02
cg18519555	5	88559798	NA	-0.0011	0.0002	1.97E-06	1.00E+00	4.36E-02
cg06985076	1	63489116	NA	-0.0014	0.0003	2.04E-06	1.00E+00	4.39E-02
cg23595667	1	116185146	*VANGL1*	0.0002	0.0000	2.09E-06	1.00E+00	4.39E-02
cg15227656	16	68303445	*SLC7A6*	-0.0016	0.0003	2.46E-06	1.00E+00	4.95E-02
cg06431702	11	66624841	*LRFN4;PC*	0.0002	0.0000	2.53E-06	1.00E+00	4.95E-02
cg09499109	15	101097659	*PRKXP1*	-0.0056	0.0012	2.55E-06	1.00E+00	4.95E-02

* Abbreviations: BMI (body mass index), CpG site (cytosine-phosphate-guanine dinucleotide sites), Chr (Chromosome), bp (base-pair), bonf_p: Bonferroni-adjusted P-value, fdr_q: False Discovery Rate-adjusted Q-value, NA (not available), SE (Standard error), EWAS (Epigenome-wide association study)

Table S3. BMI-Associated CpG Sites with FDR q < 0.05 in Meta-analysis EWAS

CpG sites	Chr	Position (bp)	Gene	Beta-coefficient	SE	bonf_P	FDR-q
cg17061862	11	9590431	*NA*	-0.0022	3.00E-04	9.84E-08	9.84E-08
cg10601624	12	6404377	*NA*	-0.0012	2.00E-04	2.60E-07	1.30E-07
cg23570810	11	315102	*IFITM1*	0.0042	6.00E-04	7.80E-06	2.60E-06
cg14951497	2	191875807	*STAT1*	0.0018	3.00E-04	2.24E-04	5.60E-05
cg10493186	1	3134756	*PRDM16*	-0.0022	4.00E-04	2.91E-04	5.75E-05
cg03038262	11	315262	*IFITM1*	0.0032	5.00E-04	3.45E-04	5.75E-05
cg15871086	18	56526595	*NA*	0.0012	2.00E-04	5.83E-04	8.33E-05
cg22930808	3	122281881	*PARP9;DTX3L*	0.0062	1.00E-03	1.17E-03	1.46E-04
cg23032421	3	3152038	*IL5RA*	-0.0014	2.00E-04	1.94E-03	2.15E-04
cg07839457	16	57023022	*NA*	0.0045	8.00E-04	2.57E-03	2.57E-04
cg08122652	3	122281939	*PARP9;DTX3L*	0.0048	8.00E-04	4.05E-03	3.69E-04
cg16704920	1	2169407	*SKI*	-0.001	2.00E-04	6.93E-03	5.77E-04
cg08926253	11	614761	*IRF7*	0.0028	5.00E-04	9.55E-03	7.34E-04
cg07833467	22	50986511	*KLHDC7B*	0.0035	6.00E-04	1.27E-02	9.04E-04
cg26950531	19	38704515	*DPF1*	-0.0025	5.00E-04	1.71E-02	1.04E-03
cg01971407	11	313624	*IFITM1*	0.0022	4.00E-04	1.76E-02	1.04E-03
cg06178669	11	63334608	*NA*	-0.0022	4.00E-04	1.76E-02	1.04E-03
cg11829870	22	50988451	*KLHDC7B*	0.0016	3.00E-04	2.11E-02	1.17E-03
cg09554443	1	167487762	*CD247*	-0.0016	3.00E-04	2.37E-02	1.25E-03

cg23401251	2	27683905	*IFT172*	-0.0014	3.00E-04	2.55E-02	1.27E-03
cg13304609	1	79085162	*IFI44L*	0.0039	7.00E-04	2.78E-02	1.32E-03
cg05883128	4	169239131	*DDX60*	0.0037	7.00E-04	2.89E-02	1.32E-03
cg16400320	8	144105210	*NA*	0.0012	2.00E-04	3.62E-02	1.57E-03
cg04907505	12	69004265	*RAP1B*	1.00E-04	0.00E+00	3.91E-02	1.60E-03
cg09026253	11	313267	*IFITM1*	0.002	4.00E-04	4.26E-02	1.60E-03
cg03607951	1	79085586	*IFI44L*	0.004	8.00E-04	4.31E-02	1.60E-03
cg08099136	6	32811251	*PSMB8;PSMB8-AS1*	0.0022	4.00E-04	4.46E-02	1.60E-03
cg12906975	8	144105259	*NA*	0.0014	3.00E-04	4.53E-02	1.60E-03
cg05478392	5	34510767	*NA*	-0.0011	2.00E-04	4.65E-02	1.60E-03
cg27395226	7	104994322	*SRPK2*	-0.0012	2.00E-04	4.98E-02	1.66E-03
cg19232929	12	52299524	*NA*	0.0011	2.00E-04	5.23E-02	1.69E-03
cg00676801	2	191876673	*STAT1*	0.0015	3.00E-04	6.82E-02	2.05E-03
cg01309328	6	32811253	*PSMB8;PSMB8-AS1*	0.002	4.00E-04	6.87E-02	2.05E-03
cg08818207	6	32820355	*TAP1*	0.0026	5.00E-04	6.99E-02	2.05E-03
cg22862003	21	42797588	*MX1*	0.0042	8.00E-04	7.48E-02	2.11E-03
cg06981309	3	146260954	*PLSCR1*	0.0037	7.00E-04	7.61E-02	2.11E-03
cg05432003	11	312518	*IFITM1*	0.0022	4.00E-04	7.92E-02	2.14E-03
cg08454563	22	31040187	*SLC35E4*	-9.00E-04	2.00E-04	8.29E-02	2.18E-03
cg16644494	22	50971601	*ODF3B*	0.0014	3.00E-04	9.49E-02	2.43E-03
cg21549285	21	42799141	*MX1*	0.006	1.20E-03	1.43E-01	3.56E-03
cg06448573	8	144107231	*NA*	8.00E-04	2.00E-04	1.68E-01	4.10E-03

cg18533225	22	50986813	*KLHDC7B*	0.0023	5.00E-04	1.99E-01	4.73E-03
cg03524147	10	119138665	*NA*	-0.0023	5.00E-04	2.14E-01	4.90E-03
cg15693572	3	22412385	*NA*	-0.0032	6.00E-04	2.16E-01	4.90E-03
cg21490635	12	13349273	*EMP1*	-7.00E-04	1.00E-04	3.62E-01	8.05E-03
cg08726900	16	89550474	*ANKRD11*	-0.002	4.00E-04	3.98E-01	8.49E-03
cg05095590	7	2139259	*MAD1L1*	0.0022	4.00E-04	3.99E-01	8.49E-03
cg26651978	17	76965407	*NA*	-0.001	2.00E-04	4.18E-01	8.56E-03
cg24304309	1	154577895	*ADAR*	0.0013	3.00E-04	4.20E-01	8.56E-03
cg16672203	6	31744391	*VWA7*	-0.0011	2.00E-04	4.48E-01	8.96E-03
cg25164589	10	63540024	*NA*	-0.0024	5.00E-04	5.10E-01	1.00E-02
cg02314339	10	91020653	*NA*	0.0013	3.00E-04	5.45E-01	1.05E-02
cg05575921	5	373378	*AHRR*	0.0033	7.00E-04	5.85E-01	1.09E-02
cg05570258	7	105662884	*CDHR3*	-0.0011	2.00E-04	5.93E-01	1.09E-02
cg12110437	8	144098888	*LY6E;LOC100133669*	0.0035	7.00E-04	5.98E-01	1.09E-02
cg18187029	12	96650848	*ELK3*	-9.00E-04	2.00E-04	6.91E-01	1.23E-02
cg11660018	11	86510915	*PRSS23*	0.0013	3.00E-04	7.08E-01	1.24E-02
cg27381930	19	33209598	*TDRD12*	5.00E-04	1.00E-04	7.69E-01	1.32E-02
cg02045948	3	32857317	*NA*	0.0016	3.00E-04	7.82E-01	1.32E-02
cg03848588	9	32525008	*DDX58*	0.001	2.00E-04	8.06E-01	1.34E-02
cg11702942	8	144102584	*LY6E*	0.0016	3.00E-04	8.47E-01	1.39E-02
cg01681525	17	79374741	*MIR4740;BAHCC1*	0.0016	3.00E-04	8.94E-01	1.42E-02
cg07928502	19	54683678	*MBOAT7*	-9.00E-04	2.00E-04	9.05E-01	1.42E-02

cg11694510	11	313354	*IFITM1*	0.0013	3.00E-04	9.06E-01	1.42E-02
cg06188083	10	91093005	*IFIT3*	0.0035	7.00E-04	9.27E-01	1.43E-02
cg08857797	17	40927699	*VPS25*	0.0012	2.00E-04	9.71E-01	1.46E-02
cg17114584	11	613792	*IRF7*	0.0024	5.00E-04	9.78E-01	1.46E-02
cg04113258	16	17527041	*XYLT1*	-6.00E-04	1.00E-04	1.00E+00	1.50E-02
cg26312951	21	42797847	*MX1*	0.0032	7.00E-04	1.00E+00	1.52E-02
cg16411857	16	57023191	*NA*	0.0016	3.00E-04	1.00E+00	1.56E-02
cg15065340	3	195632915	*TNK2*	0.0024	5.00E-04	1.00E+00	1.83E-02
cg21393163	1	12217629	*NA*	0.001	2.00E-04	1.00E+00	1.89E-02
cg24755459	14	101908865	*NA*	-6.00E-04	1.00E-04	1.00E+00	2.07E-02
cg11224765	22	50971109	*ODF3B*	0.0022	5.00E-04	1.00E+00	2.10E-02
cg18745507	19	10415557	*ZGLP1*	-0.001	2.00E-04	1.00E+00	2.10E-02
cg12593793	1	156074135	*LMNA*	-0.0012	3.00E-04	1.00E+00	2.20E-02
cg11791770	11	611791	*PHRF1*	9.00E-04	2.00E-04	1.00E+00	2.28E-02
cg15530112	14	53929908	*NA*	9.00E-04	2.00E-04	1.00E+00	2.44E-02
cg22910549	22	50985117	*KLHDC7B*	0.0021	5.00E-04	1.00E+00	2.51E-02
cg25125231	12	630773	*B4GALNT3*	0.001	2.00E-04	1.00E+00	2.51E-02
cg14392283	8	144103587	*LY6E;LY6E*	0.0022	5.00E-04	1.00E+00	2.51E-02
cg22282590	19	17514117	*BST2*	6.00E-04	1.00E-04	1.00E+00	2.72E-02
cg04304036	16	68334619	*SLC7A6;SLC7A6OS*	0.0011	2.00E-04	1.00E+00	2.84E-02
cg04927537	17	76976091	*LGALS3BP*	0.0024	5.00E-04	1.00E+00	2.89E-02
cg10552523	11	313478	*IFITM1*	0.0023	5.00E-04	1.00E+00	2.89E-02

cg27430977	19	1826373	*REXO1*	-0.001	2.00E-04	1.00E+00	2.89E-02
cg20545410	10	33232548	*ITGB1*	-0.0012	3.00E-04	1.00E+00	3.02E-02
cg12836643	22	39129370	*GTPBP1*	-0.001	2.00E-04	1.00E+00	3.02E-02
cg21240420	6	33148096	*COL11A2*	-8.00E-04	2.00E-04	1.00E+00	3.02E-02
cg17178761	17	55682851	*LOC101927539;MSI2*	-0.0014	3.00E-04	1.00E+00	3.08E-02
cg20318217	2	147172982	*NA*	0.0011	3.00E-04	1.00E+00	3.08E-02
cg13315690	17	1944831	*DPH1;OVCA2*	-9.00E-04	2.00E-04	1.00E+00	3.08E-02
cg19459791	15	65363022	*NA*	-9.00E-04	2.00E-04	1.00E+00	3.17E-02
cg01176028	21	43653234	*ABCG1*	0.0013	3.00E-04	1.00E+00	3.20E-02
cg14750551	3	122401343	*PARP14*	0.0015	3.00E-04	1.00E+00	3.20E-02
cg12424383	1	27990967	*NA*	0.001	2.00E-04	1.00E+00	3.20E-02
cg02032125	14	101000186	*NA*	-0.001	2.00E-04	1.00E+00	3.33E-02
cg01286133	21	27540106	*APP*	5.00E-04	1.00E-04	1.00E+00	3.35E-02
cg18763536	12	11812062	*ETV6*	-0.0012	3.00E-04	1.00E+00	3.37E-02
cg02782510	3	160820084	*B3GALNT1*	-7.00E-04	2.00E-04	1.00E+00	3.37E-02
cg02346342	8	74332435	*STAU2-AS1*	0.0011	3.00E-04	1.00E+00	3.40E-02
cg25112191	1	151804260	*RORC*	0.001	2.00E-04	1.00E+00	3.50E-02
cg00401463	10	133978450	*JAKMIP3*	0.002	5.00E-04	1.00E+00	3.64E-02
cg19025187	3	195808255	*TFRC*	6.00E-04	1.00E-04	1.00E+00	3.64E-02
cg21040575	17	6921465	*MIR497HG;MIR195*	-0.001	2.00E-04	1.00E+00	3.64E-02
cg01329690	21	38580129	*DSCR9*	0.0012	3.00E-04	1.00E+00	3.64E-02
cg23716690	1	10314029	*KIF1B*	-0.001	2.00E-04	1.00E+00	3.64E-02

cg01028142	2	7004578	*CMPK2*	0.003	7.00E-04	1.00E+00	3.64E-02
cg05021589	6	6588931	*LY86;LY86-AS1*	0.0012	3.00E-04	1.00E+00	3.99E-02
cg14341551	9	139913237	*ABCA2*	-7.00E-04	1.00E-04	1.00E+00	4.16E-02
cg02863179	10	63779053	*ARID5B*	-0.0012	3.00E-04	1.00E+00	4.18E-02
cg26033520	10	74004071	*NA*	0.0013	3.00E-04	1.00E+00	4.22E-02
cg11594303	9	123639439	*PHF19*	1.00E-04	0.00E+00	1.00E+00	4.22E-02
cg21440365	8	130995997	*FAM49B*	-6.00E-04	1.00E-04	1.00E+00	4.22E-02
cg03358636	3	197474006	*KIAA0226*	0.0013	3.00E-04	1.00E+00	4.22E-02
cg22016995	11	614787	*IRF7*	0.0022	5.00E-04	1.00E+00	4.31E-02
cg14293575	22	18635460	*USP18*	0.003	7.00E-04	1.00E+00	4.33E-02
cg17778165	20	10414372	*MKKS*	9.00E-04	2.00E-04	1.00E+00	4.54E-02
cg03587597	8	144105055	*NA*	8.00E-04	2.00E-04	1.00E+00	4.54E-02
cg11799593	12	68845869	*LOC100507195*	0.0044	1.00E-03	1.00E+00	4.67E-02
cg19413066	17	76965347	*NA*	-0.0011	2.00E-04	1.00E+00	4.67E-02
cg13859208	12	130649720	*FZD10;FZD10*	-0.0017	4.00E-04	1.00E+00	4.74E-02
cg14870271	17	76976010	*LGALS3BP*	0.0019	4.00E-04	1.00E+00	4.75E-02
cg13039251	5	32018601	*PDZD2*	-0.0012	3.00E-04	1.00E+00	4.87E-02

* Abbreviations: BMI (body mass index), CpG site (cytosine-phosphate-guanine dinucleotide sites), Chr (Chromosome), bp (base-pair), bonf_p: Bonferroni-adjusted P-value, fdr_q: False Discovery Rate-adjusted Q-value, SE (Standard error), EWAS (Epigenome-wide association study)

Table S4. BMI-Associated CpG Sites with FDR q < 0.05 in Meta-analysis EWAS after adjusting for sCD14.

CpG sites	Chr	Position (bp)	Gene	Beta-coefficient	SE	p-value	bonf_P	fdr_q
cg17061862	11	9590431	NA	-0.0022	0.0003	6.44E-11	2.41E-05	2.41E-05
cg10601624	12	6404377	NA	-0.0011	0.0002	3.20E-10	1.20E-04	5.99E-05
cg04907505	12	69004265	*RAP1B*	0.0001	0.0000	9.68E-09	3.63E-03	1.21E-03
cg06178669	11	63334608	NA	-0.0024	0.0004	6.41E-08	2.40E-02	6.01E-03
cg23032421	3	3152038	*IL5RA*	-0.0013	0.0003	2.72E-07	1.02E-01	1.88E-02
cg26950531	19	38704515	*DPF1*	-0.0026	0.0005	3.05E-07	1.14E-01	1.88E-02
cg23570810	11	315102	*IFITM1*	0.0034	0.0007	3.71E-07	1.39E-01	1.88E-02
cg05570258	7	105662884	*CDHR3*	-0.0013	0.0003	4.31E-07	1.61E-01	1.88E-02
cg23401251	2	27683905	*IFT172*	-0.0015	0.0003	4.57E-07	1.71E-01	1.88E-02
cg08454563	22	31040187	*SLC35E4*	-0.0009	0.0002	5.03E-07	1.88E-01	1.88E-02
cg10493186	1	3134756	*PRDM16*	-0.0019	0.0004	1.25E-06	4.67E-01	4.24E-02
cg21490635	12	13349273	*EMP1*	-0.0008	0.0002	1.52E-06	5.69E-01	4.60E-02
cg11799593	12	68845869	*LOC100507195*	0.0054	0.0011	1.60E-06	5.98E-01	4.60E-02
cg20545410	10	33232548	*ITGB1*	-0.0014	0.0003	1.91E-06	7.16E-01	5.00E-02
cg05095590	7	2139259	*MAD1L1*	0.0023	0.0005	2.00E-06	7.50E-01	5.00E-02

* Abbreviations: BMI (body mass index), CpG site (cytosine-phosphate-guanine dinucleotide sites), Chr (Chromosome), bp (base-pair), bonf_p: Bonferroni-adjusted P-value, fdr_q: False Discovery Rate-adjusted Q-value, NA (not available), SE (Standard error), EWAS (Epigenome-wide association study).

Table S5. Comparison of Effect Sizes and p-values Before and After Adjusting sCD14.

	Before			After		
CpG site	Beta-coefficient	SE	p-value	Beta-coefficients	SE	p-value
cg17061862	-0.21991	0.03007	2.63E-13	-0.2158	0.033	6.44E-11
cg10601624	-0.11773	0.0164	6.95E-13	-0.1132	0.018	3.20E-10
cg23570810	0.41917	0.06256	2.08E-11	0.3386	0.0666	3.71E-07
cg14951497	0.18047	0.02915	5.98E-10	0.1432	0.0314	5.05E-06
cg10493186	-0.21579	0.03509	7.77E-10	-0.186	0.0384	1.25E-06
cg03038262	0.32301	0.05276	9.22E-10	0.2485	0.0561	9.40E-06
cg15871086	0.11935	0.01977	1.56E-09	0.1001	0.0213	2.48E-06
cg22930808	0.61637	0.10403	3.13E-09	0.5008	0.1122	8.11E-06
cg23032421	-0.13711	0.02347	5.17E-09	-0.1342	0.0261	2.72E-07
cg07839457	0.45253	0.0781	6.86E-09	0.3534	0.0834	2.24E-05
cg08122652	0.47864	0.08372	1.08E-08	0.3666	0.0903	4.87E-05
cg16704920	-0.10271	0.01826	1.85E-08	-0.0924	0.0203	5.50E-06
cg08926253	0.28066	0.05039	2.55E-08	0.2224	0.0546	4.55E-05
cg07833467	0.35278	0.0639	3.38E-08	0.2863	0.0698	4.13E-05
cg26950531	-0.25356	0.04638	4.57E-08	-0.2608	0.0509	3.05E-07
cg01971407	0.21961	0.0402	4.69E-08	0.1741	0.0432	5.49E-05
cg06178669	-0.22064	0.0404	4.71E-08	-0.2427	0.0449	6.41E-08
cg11829870	0.16065	0.02959	5.65E-08	0.1395	0.0323	1.61E-05
cg09554443	-0.1561	0.02886	6.34E-08	-0.1408	0.0314	7.49E-06
cg23401251	-0.14451	0.02678	6.81E-08	-0.1494	0.0296	4.57E-07
cg13304609	0.38655	0.07184	7.42E-08	0.3087	0.0781	7.78E-05
cg05883128	0.3656	0.06804	7.73E-08	0.3035	0.0728	3.04E-05
cg16400320	0.12049	0.02259	9.66E-08	0.0985	0.0246	6.45E-05
cg04907505	0.01036	0.00195	1.04E-07	0.0117	0.002	9.68E-09

cg09026253	0.20177	0.03805	1.14E-07	0.1621	0.0416	9.83E-05
cg03607951	0.39782	0.07504	1.15E-07	0.3015	0.0811	0.0002
cg08099136	0.21878	0.04132	1.19E-07	0.1852	0.0448	3.50E-05
cg12906975	0.14126	0.02669	1.21E-07	0.1148	0.0292	8.36E-05
cg05478392	-0.11338	0.02145	1.24E-07	-0.0766	0.0219	0.000479
cg27395226	-0.1196	0.02267	1.33E-07	-0.1158	0.025	3.72E-06

* Abbreviations: CpG site (cytosine-phosphate-guanine dinucleotide sites), bp (base-pair), SE (Standard error), Beta-coefficients were reported as coefficient ×100, so as well for SE.

Table S6. Comparative Analysis of BMI-Associated CpG Sites Between Meta-Analysis of EWAS and the WHI Cohort.

				Meta-Analysis of EWAS			WHI Cohort		
**CpG sites**	**Chr**	**Position (bp)**	**Gene**	**Beta-coefficient**	**SE**	**p-value**	**Beta-coefficients**	**SE**	**p-value**
cg00574958	11	68607622	*CPT1A*	-0.12328	0.03175	0.0001033	-0.000650544	4.91E-05	4.57E-40
cg17501210	6	166970252	*RPS6KA2*	0.01073	0.04474	0.8104	-0.001760659	0.00014823	1.55E-32
cg01130991	17	46510392	NA	0.04131	0.02119	0.05127	0.001006066	9.35E-05	5.54E-27
cg17901584	1	55353706	*DHCR24*	-0.05045	0.03808	0.1852	-0.001230891	0.00011888	4.02E-25
cg19750657	13	38935967	*UFM1*	0.0253	0.03351	0.4504	0.001170028	0.00011618	7.45E-24
cg12992827	3	101901234	NA	0.04288	0.02919	0.1418	-0.001157125	0.00011541	1.17E-23
cg06192883	15	52554171	*MYO5C*	0.00284	0.03145	0.9281	0.001130768	0.00011344	2.10E-23
cg18181703	17	76354621	*SOCS3*	0.09887	0.03371	0.003356	-0.001293723	0.00012987	2.24E-23
cg24174557	17	57903544	*VMP1*	-0.01125	0.03704	0.7613	-0.001197589	0.00012181	8.23E-23
cg13222915	1	184598594	*NA*	-0.0019	0.02032	0.9255	-0.000786548	8.20E-05	8.52E-22
cg13123009	6	31681882	*LY6G6D;LY6G6E*	0.0198	0.01893	0.2954	0.000800121	8.35E-05	9.68E-22
cg03358636	3	197474006	*KIAA0226*	0.1281	0.02938	1.30E-05	0.001072885	0.00011249	1.46E-21
cg10927968	11	1807333	NA	0.10212	0.03545	0.003967	0.000967853	0.00010351	8.73E-21
cg04816311	7	1066650	*C7orf50*	0.1127	0.03864	0.003539	0.00114308	0.00012538	7.70E-20
cg17058475	11	68607737	*CPT1A*	-0.07272	0.03625	0.04488	-0.000656733	7.21E-05	8.23E-20

cg27243685	21	43642366	*ABCG1*	0.0646	0.01951	0.0009291	0.000567415	6.26E-05	1.31E-19
cg09831562	3	181327125	*SOX2-OT*	0.00077	0.02884	0.9787	0.00109385	0.00012075	1.32E-19
cg06898549	12	41083590	NA	0.04378	0.03728	0.2402	0.001193395	0.00013278	2.52E-19
cg02650017	17	47301614	*PHOSPHO1*	-0.00431	0.01748	0.8052	-0.000284266	3.19E-05	5.24E-19
cg26663590	16	28959310	NA	0.02086	0.02556	0.4145	0.00089617	0.00010105	7.39E-19
cg01101459	1	234871477	NA	0.02149	0.03273	0.5114	0.00076785	8.73E-05	1.44E-18
cg06559575	12	53490352	*IGFBP6*	-0.02158	0.02149	0.3153	-0.000702687	8.01E-05	1.83E-18
cg26955383	10	105218660	*CALHM1*	-0.02498	0.02704	0.3555	0.000850859	9.79E-05	3.52E-18
cg03940776	6	158490013	*SYNJ2*	-0.02908	0.01732	0.09318	-0.000614586	7.18E-05	1.13E-17
cg03318904	22	39801522	*TAB1*	0.02206	0.02122	0.2984	0.000607905	7.15E-05	1.95E-17
cg25130381	1	27440721	*SLC9A1*	-0.0309	0.02086	0.1386	0.000654088	7.72E-05	2.43E-17
cg12269535	6	43142014	*SRF*	0.02203	0.02486	0.3755	-0.000987158	0.00011804	6.11E-17
cg07960624	8	119208486	*SAMD12*	-0.06721	0.04512	0.1363	-0.001218383	0.00014733	1.34E-16
cg13274938	17	38493822	*RARA*	0.00806	0.01572	0.608	0.00056461	6.84E-05	1.49E-16
cg12593793	1	156074135	*LMNA*	-0.12147	0.02647	4.46E-06	-0.000722981	8.76E-05	1.58E-16
cg13134297	7	30737556	*CRHR2*	-0.04526	0.02959	0.1262	-0.000728003	8.88E-05	2.41E-16
cg07136133	11	36422377	*PRR5L*	-0.04134	0.02448	0.09121	-0.000717829	8.86E-05	5.32E-16
cg06568880	17	2166583	*SMG6*	0.01456	0.0123	0.2364	0.000434752	5.37E-05	5.50E-16
cg23866916	19	1155738	*SBNO2*	0.01559	0.01494	0.2965	-0.000507014	6.31E-05	9.02E-16
cg26804423	7	8201134	*ICA1*	-0.00377	0.01939	0.8458	0.000560645	6.98E-05	9.45E-16

* Abbreviations: BMI: body mass index. CpG sites: Cytosine-phosphate-Guanine sites. Chr: Chromosome. Position (bp): Position in base pairs on the chromosome. Beta-coefficient: The estimated effect size of the CpG site on BMI. SE: Standard Error (of the beta-coefficient). fdr_q: False Discovery Rate-adjusted Q-value. EWAS: Epigenome-Wide Association Study. NA: not available. WHI: Women's Health Initiative.

Table S7. Gene Ontology Terms Enriched in Biological Process Categories with Statistical Significance.

ONTOLOGY	TERM	N	DE	P.DE	FDR
GO:0051607	BP	defense response to virus	289	8	4.22E-11
GO:0140546	BP	defense response to symbiont	290	8	4.24E-11
GO:0009615	BP	response to virus	393	8	7.41E-10
GO:0006955	BP	immune response	1604	11	8.83E-09
GO:0140888	BP	interferon-mediated signaling pathway	88	5	1.03E-08
GO:0098542	BP	defense response to other organism	1103	9	5.63E-08
GO:0002376	BP	immune system process	2383	12	7.72E-08
GO:0045087	BP	innate immune response	868	8	1.69E-07
GO:0006952	BP	defense response	1715	10	2.21E-07
GO:0060337	BP	type I interferon-mediated signaling pathway	65	4	2.78E-07
GO:0071357	BP	cellular response to type I interferon	66	4	2.82E-07
GO:0034340	BP	response to type I interferon	72	4	4.43E-07
GO:0051707	BP	response to other organism	1425	9	7.13E-07
GO:0043207	BP	response to external biotic stimulus	1428	9	7.22E-07
GO:0009607	BP	response to biotic stimulus	1466	9	9.53E-07
GO:0019221	BP	cytokine-mediated signaling pathway	472	6	1.58E-06
GO:0060333	BP	type II interferon-mediated signaling pathway	25	3	1.76E-06
GO:0002230	BP	positive regulation of defense response to virus by host	31	3	1.98E-06
GO:0002831	BP	regulation of response to biotic stimulus	454	6	2.01E-06
GO:0044419	BP	biological process involved in interspecies interaction between organisms	1593	9	2.26E-06

* Abbreviations: BP: Biological Process, one of the categories of Gene Ontology that describes processes accomplished by one or more ordered assemblies of molecular functions. N: Number of genes involved in the GO term. DE: Differentially Expressed, indicating the number of genes that show a significant difference in expression level in the condition being studied. P.DE: P-value for Differentially Expressed, which is the probability that the observed differential expression is due to chance. FDR: False Discovery Rate, a statistical method used in multiple hypothesis testing to correct for the problem of Type I errors.

Figure S1: 450K EWAS for Body Mass Index.

a) Plot a is the QQ plot for the unadjusted p-values. b) Plot b is the Manhattan plot for the unadjusted p-values.

Plot a and plot b models were adjusted for covariates: age, hepatitis C virus infection, diabetes status, smoking status, HIV viral load, and computed cell-type proportions.

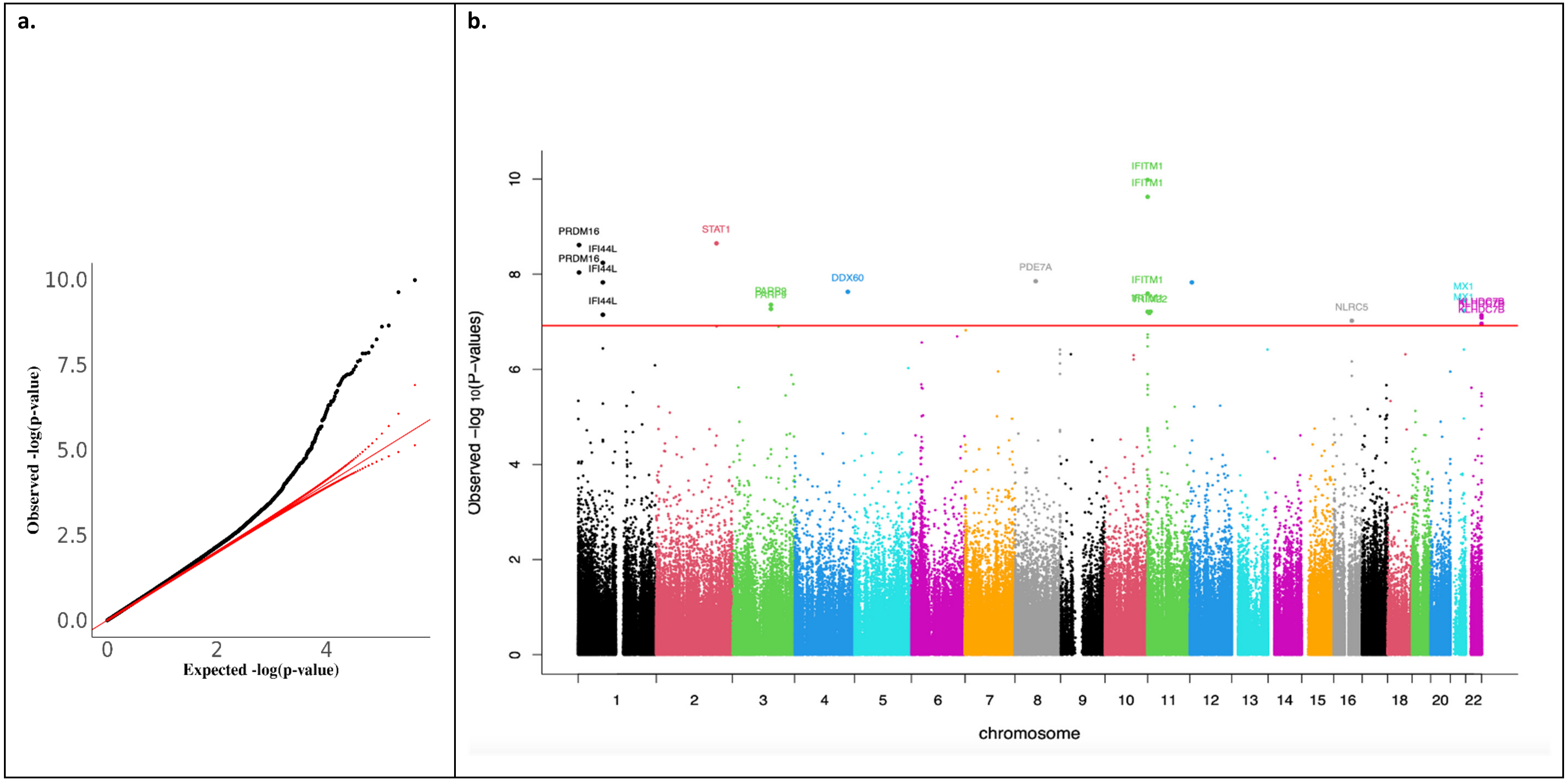

Figure S2: Results for the 850K EWAS for Body Mass Index.

a) Plot a is the QQ-plot for the adjusted p-values using Bacon, inflation factor (IF)=0.979.

b) Plot b is the Manhattan plot for the adjusted p-values using Bacon.

c) Plot c is the QQ-plot for the unadjusted p-values, IF=0.773.

d) Plot d is the Manhattan plot for the unadjusted p-values.

Plot a and b models were adjusted for covariates: age, hepatitis C virus infection, smoking status, HIV viral load, and computed cell-type proportions.

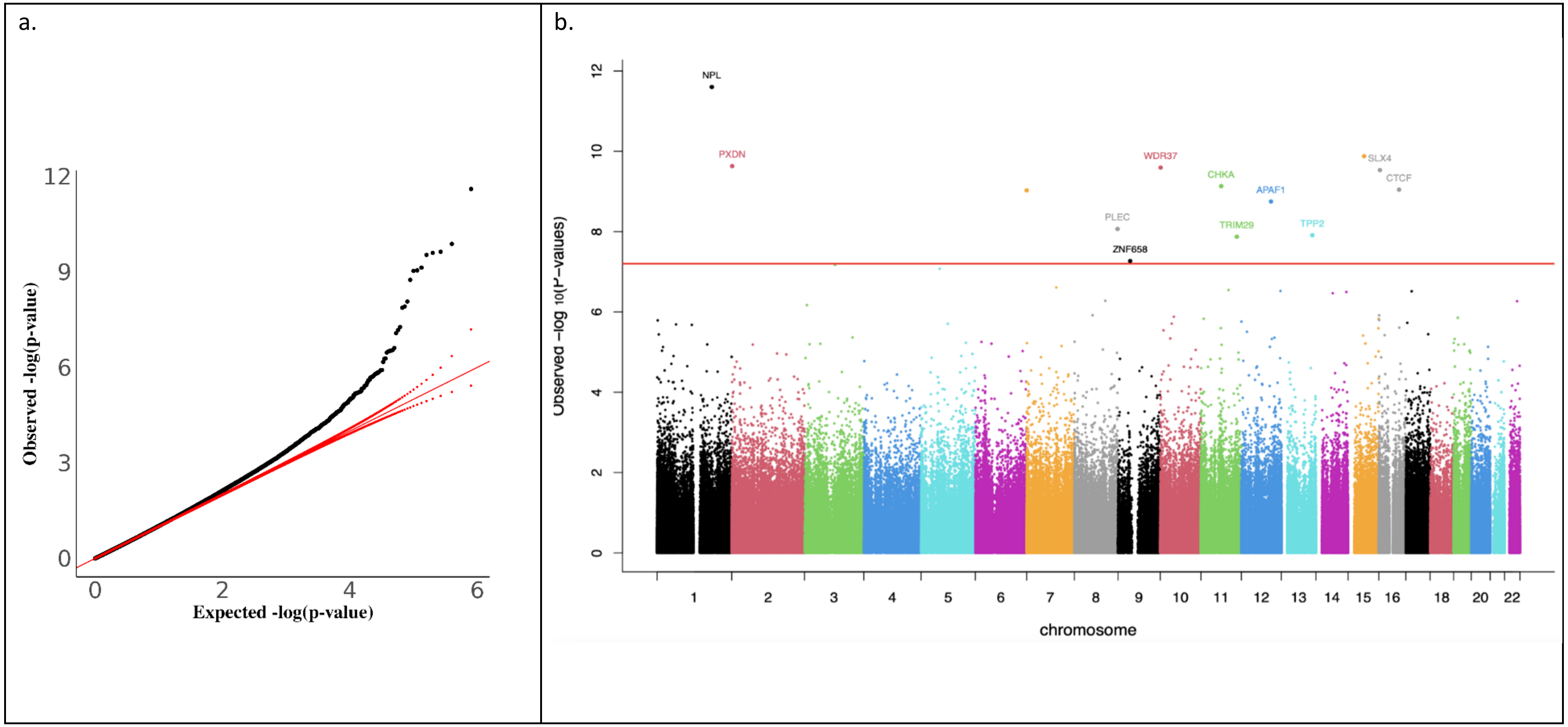

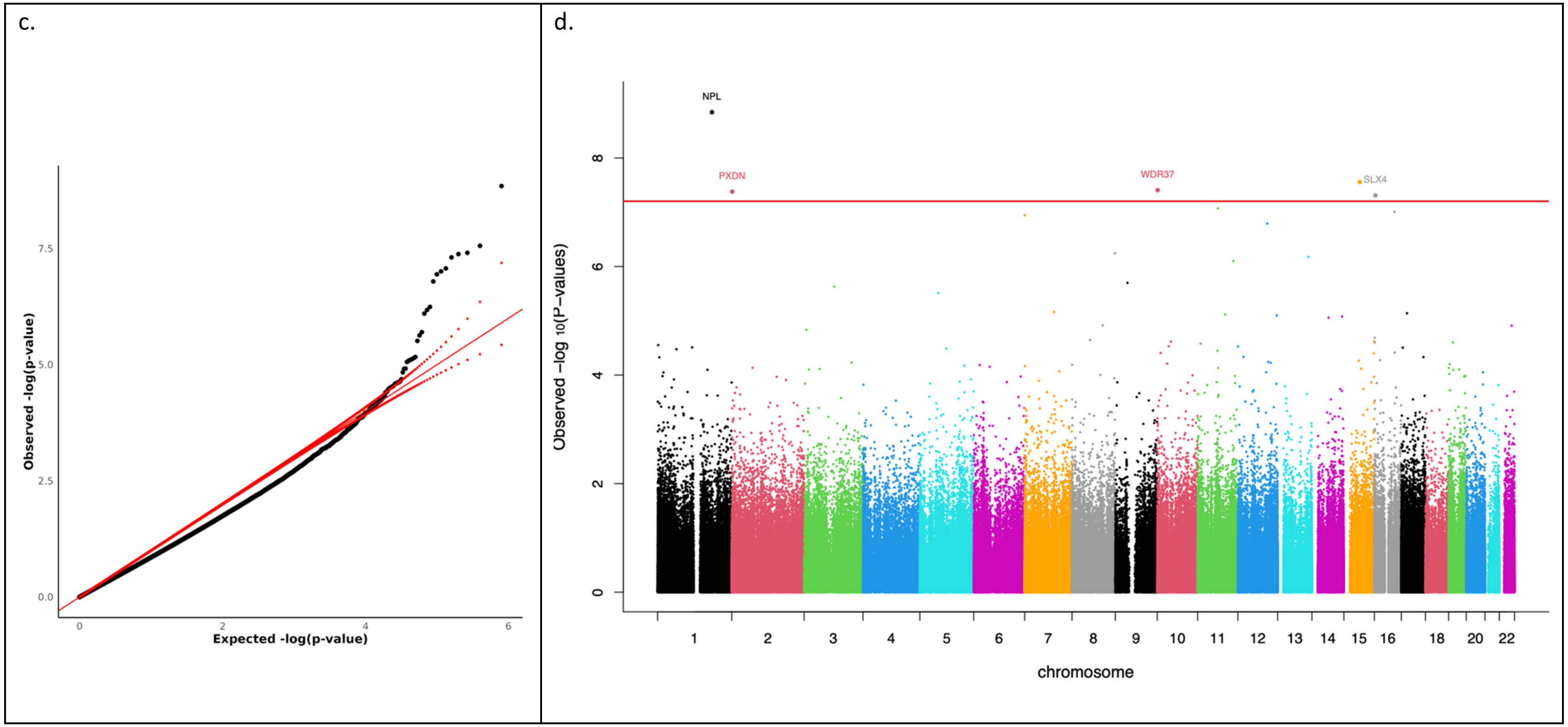
c.
Observed -log(p-value)
Expected -log(p-value)
d.
NPL
PXDN
WDR37
SLX4
Observed −log 10(P−values)
chromosome

Figure S3: Results for the 450K EWAS for Body Mass Index after adjusting for sCD14.

a) Plot a: QQ-plot for the unadjusted p-values.

b) Plot b: Manhattan plot for the unadjusted p-values.

Models were adjusted for covariates: sCD14 level, age, hepatitis C virus infection, diabetes status, smoking status, HIV viral load, and computed cell-type proportions.

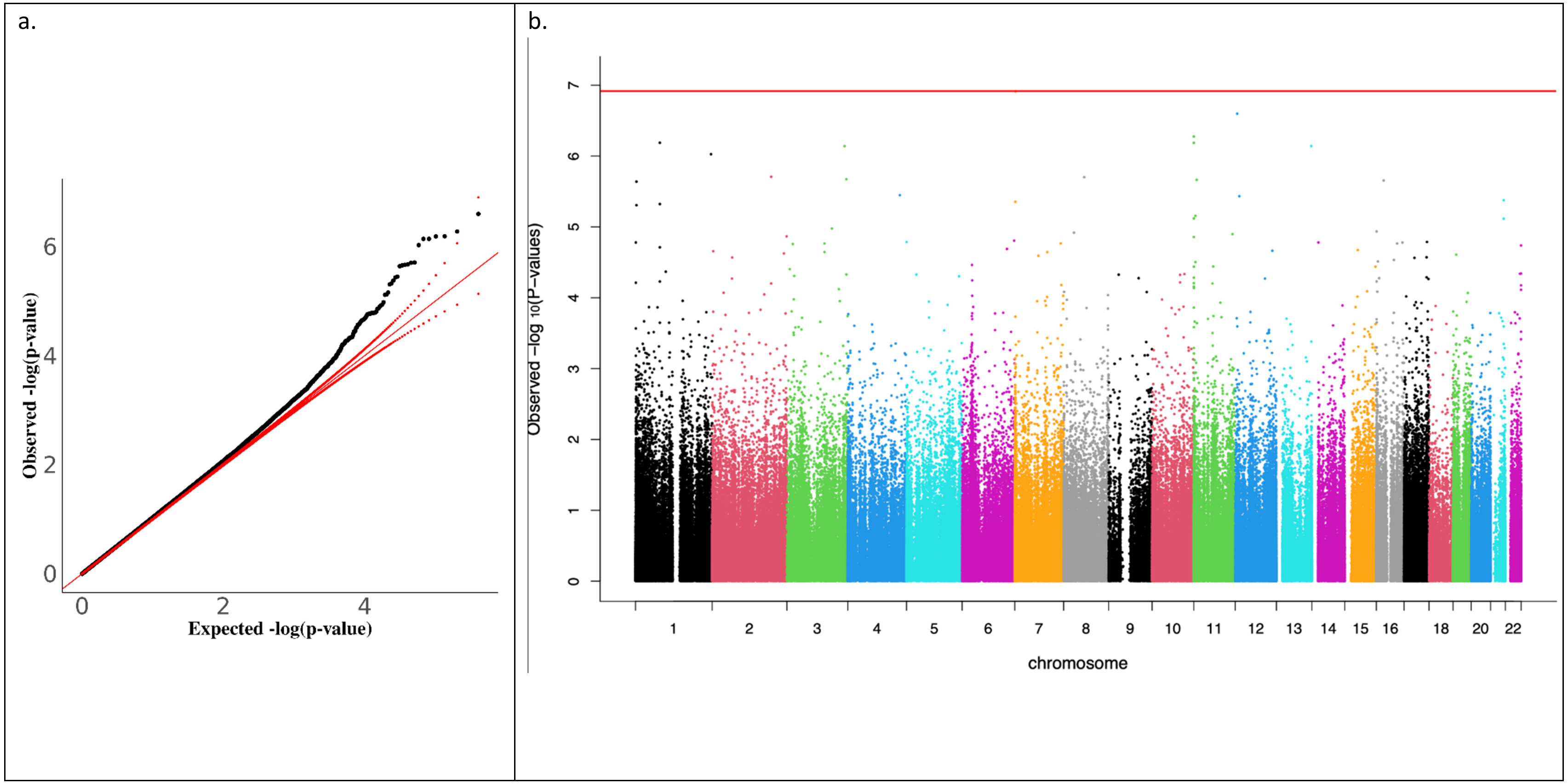

Figure S4: Results for the 850K EWAS for Body Mass Index after adjusting for sCD14.

a) Plot c is the QQ-plot for the adjusted p-values using Bacon.

b) Plot d is the Manhattan plot for adjusted p-values using Bacon.

Models were adjusted for covariates: sCD14 levels, age, hepatitis C virus infection, diabetes status, smoking status, HIV viral load, and computed cell-type proportions.

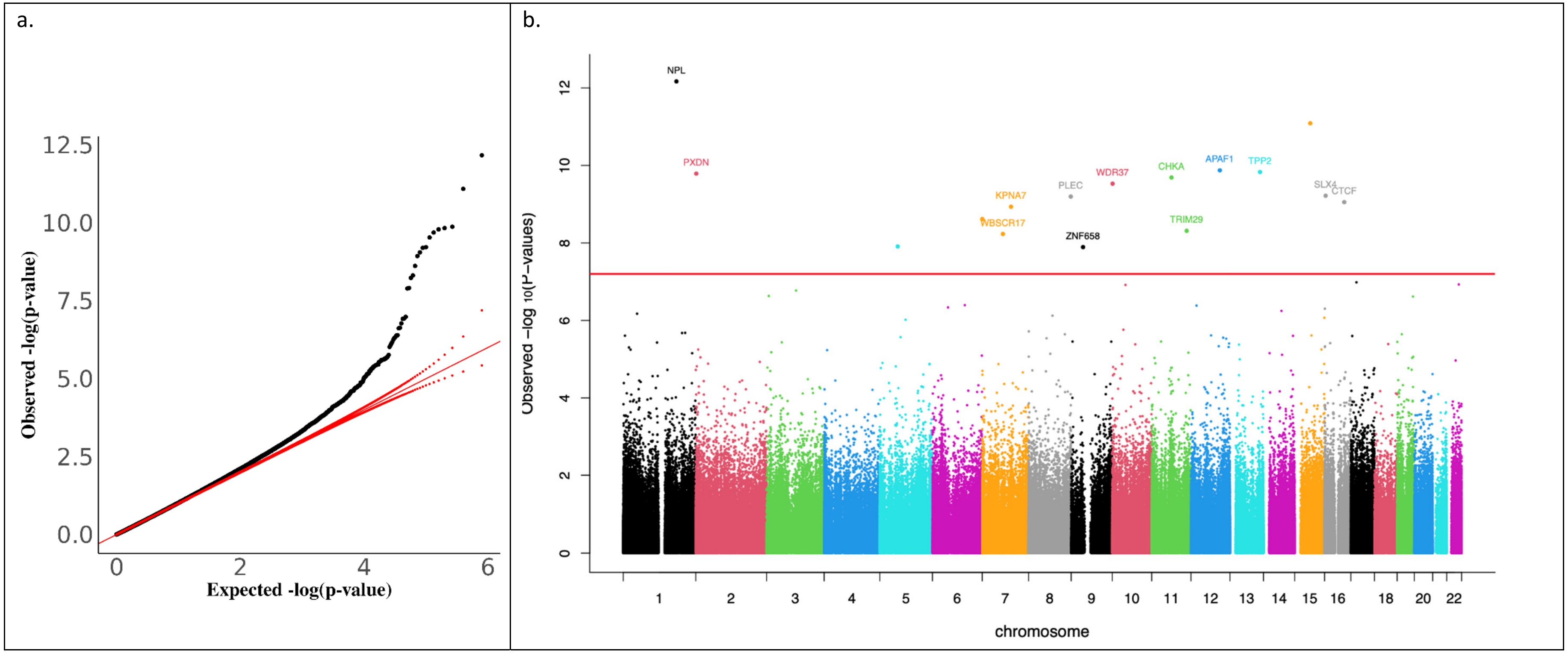

Figure S5. Regional plots of significant CpG sites associated with body mass index from meta-analysis among African Americans in Veteran Aging Cohort Study.

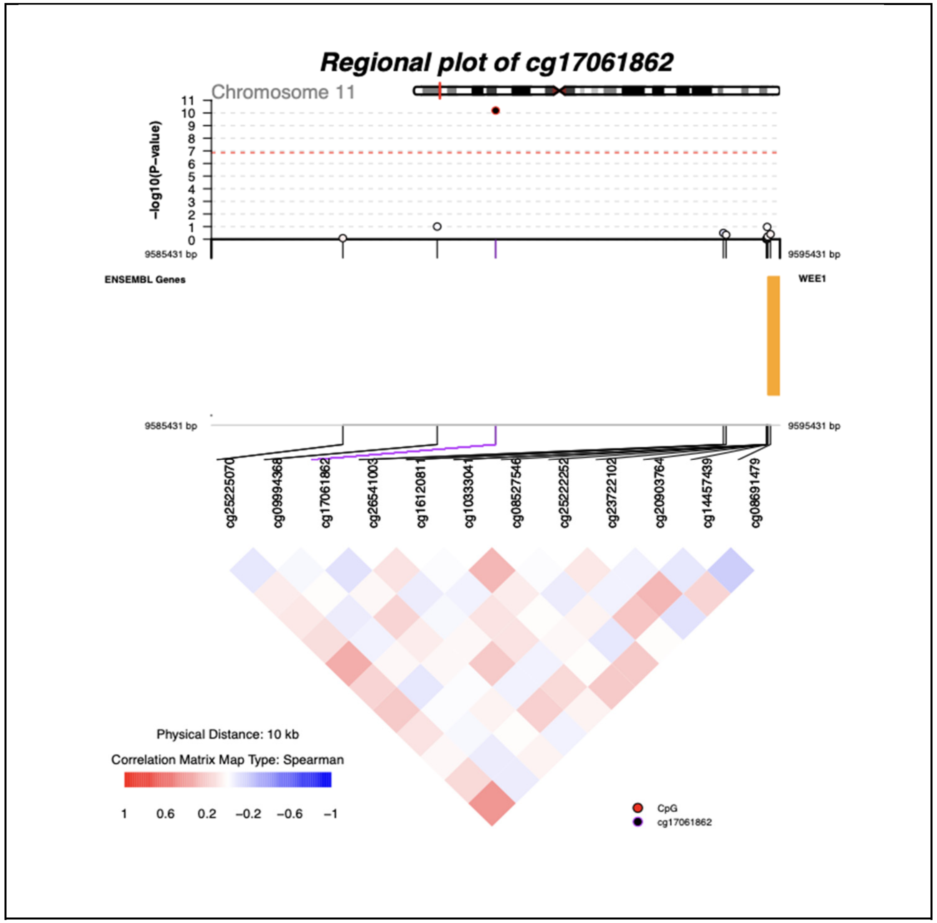

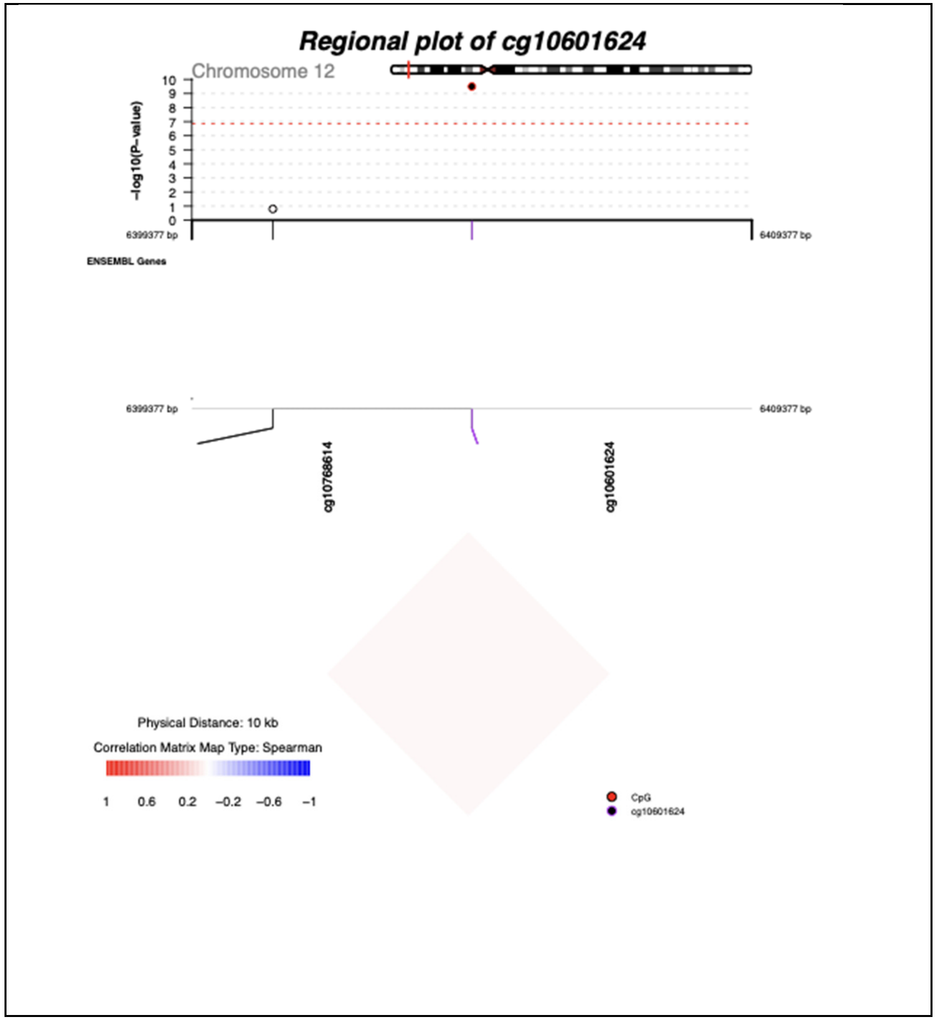

Regional plot of cg10601624
Chromosome 12
-log10(P-value)
6399377 bp
6409377 bp
ENSEMBL Genes
cg10768614
cg10601624
Physical Distance: 10 kb
Correlation Matrix Map Type: Spearman
1
0.6
0.2
−0.2
−0.6
−1
CpG
cg10601624

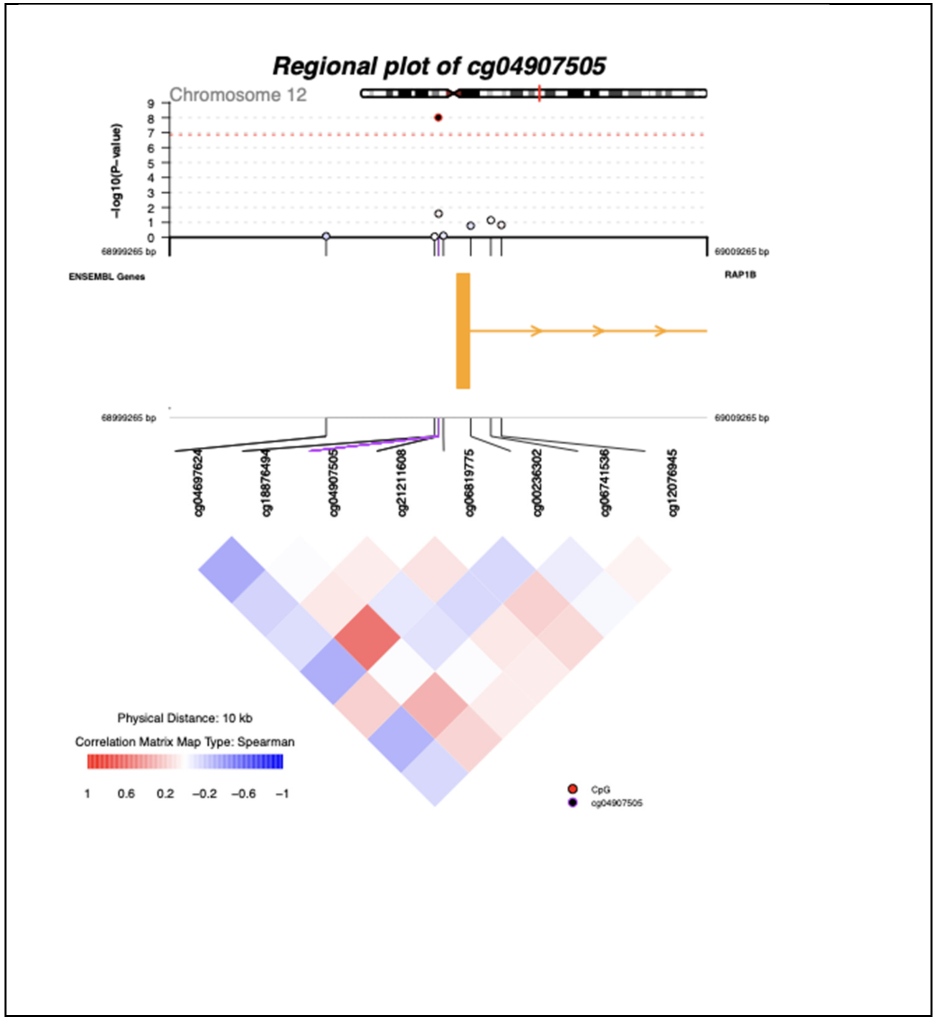

Regional plot of cg04907505
Chromosome 12
−log10(P−value)
68999265 bp
69009265 bp
ENSEMBL Genes
RAP1B
cg04697624
cg18876494
cg04907505
cg21211608
cg06819775
cg00236302
cg06741536
cg12076945
Physical Distance: 10 kb
Correlation Matrix Map Type: Spearman
1 0.6 0.2 −0.2 −0.6 −1
CpG
cg04907505

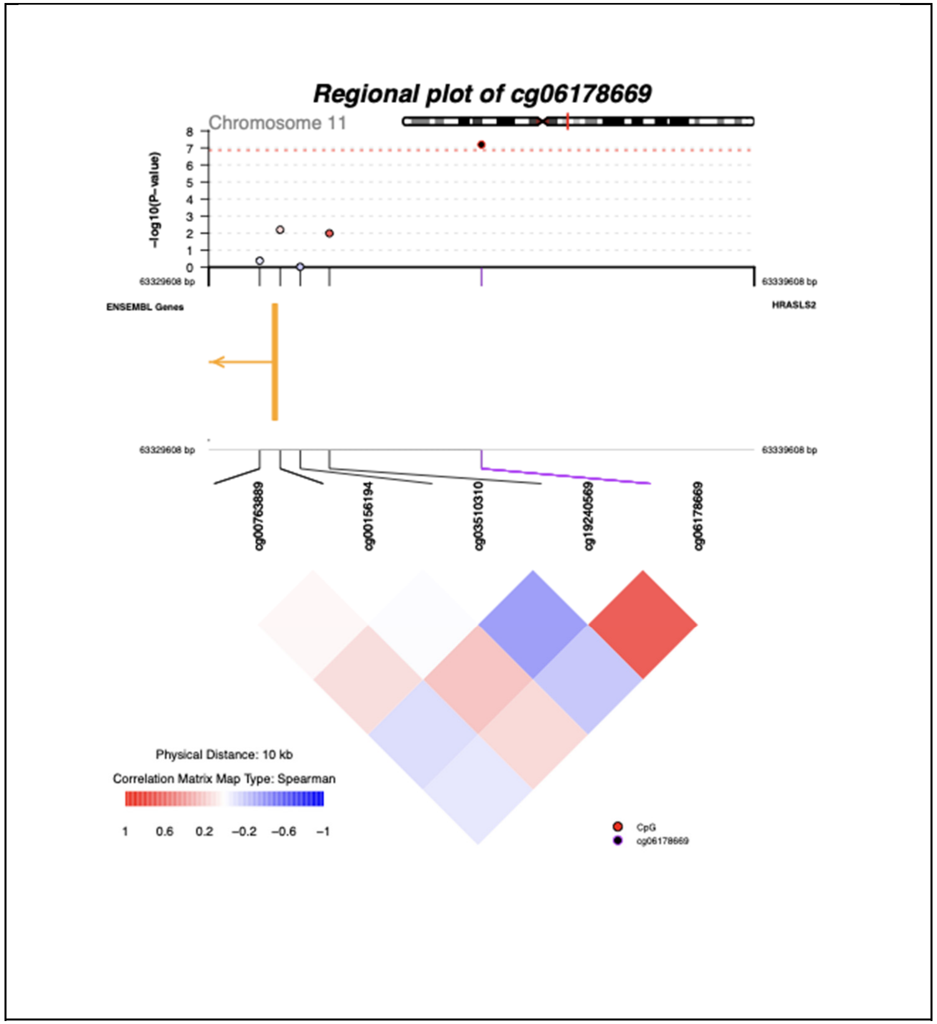
Regional plot of cg06178669
Chromosome 11
−log10(P−value)
63329608 bp
63339608 bp
ENSEMBL Genes
HRASLS2
cg00763889
cg00156194
cg03510310
cg19240569
cg06178669
Physical Distance: 10 kb
Correlation Matrix Map Type: Spearman
1 0.6 0.2 −0.2 −0.6 −1
CpG
cg06178669

Figure S6. Methylation risk score for BMI calculated using 349 CpG regression coefficients and VACS DNA methylation beta values.

*** Abbreviations: BMI: body mass index. CpG sites: Cytosine-phosphate-Guanine sites. VACS: Veterans Aging Cohort Study.**

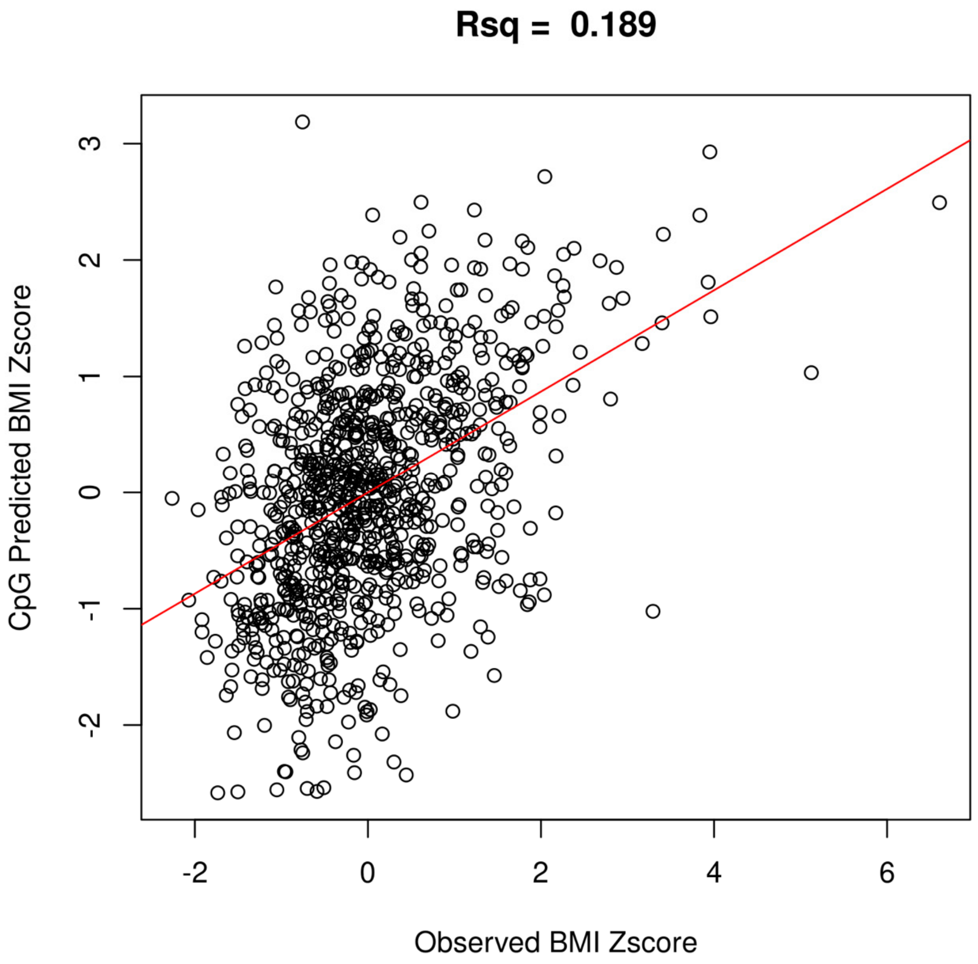

Figure S7. Cross-Validation of Predictive Accuracy for BMI Variance Using Basic Elastic-Net Model on CpG Sites in PWH

BMI: Body Mass Index. CpG: Cytosine-phosphate-Guanine site. MRS: Methylation Risk Score. A score that represents the cumulative effect of methylation at multiple CpG sites on a trait or disease risk, such as BMI. PWH: People With HIV. VACS: Veterans Aging Cohort Study. Rsq or R^2: Coefficient of Determination.

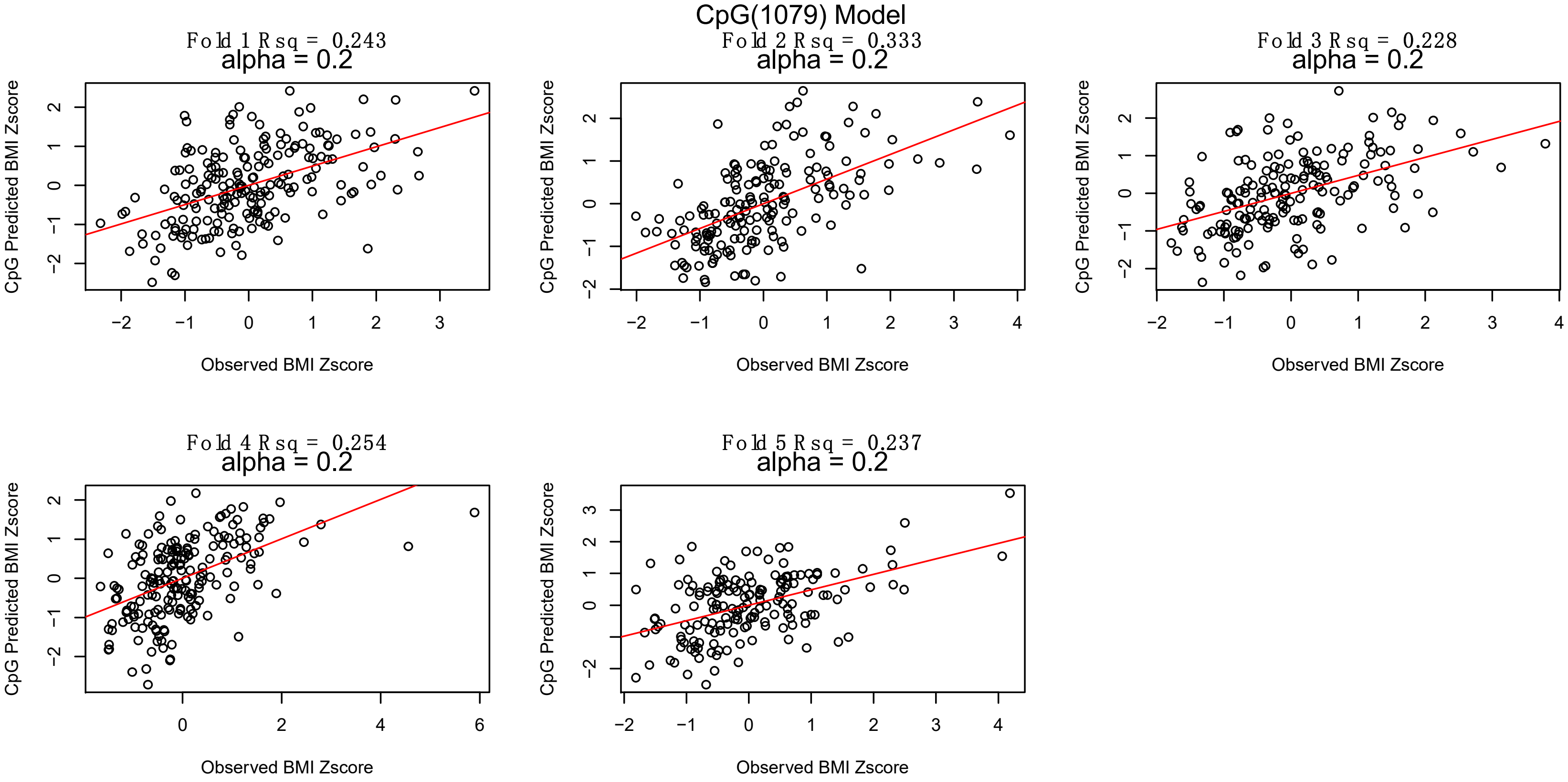

Figure S8. Cross-Validation of Predictive Accuracy for BMI Variance Using Elastic-Net Models Incorporating Age and Smoking on CpG Sites in PWH

BMI: Body Mass Index. CpG: Cytosine-phosphate-Guanine site. MRS: Methylation Risk Score. A score that represents the cumulative effect of methylation at multiple CpG sites on a trait or disease risk, such as BMI. PWH: People With HIV. VACS: Veterans Aging Cohort Study. Rsq or R^2: Coefficient of Determination.

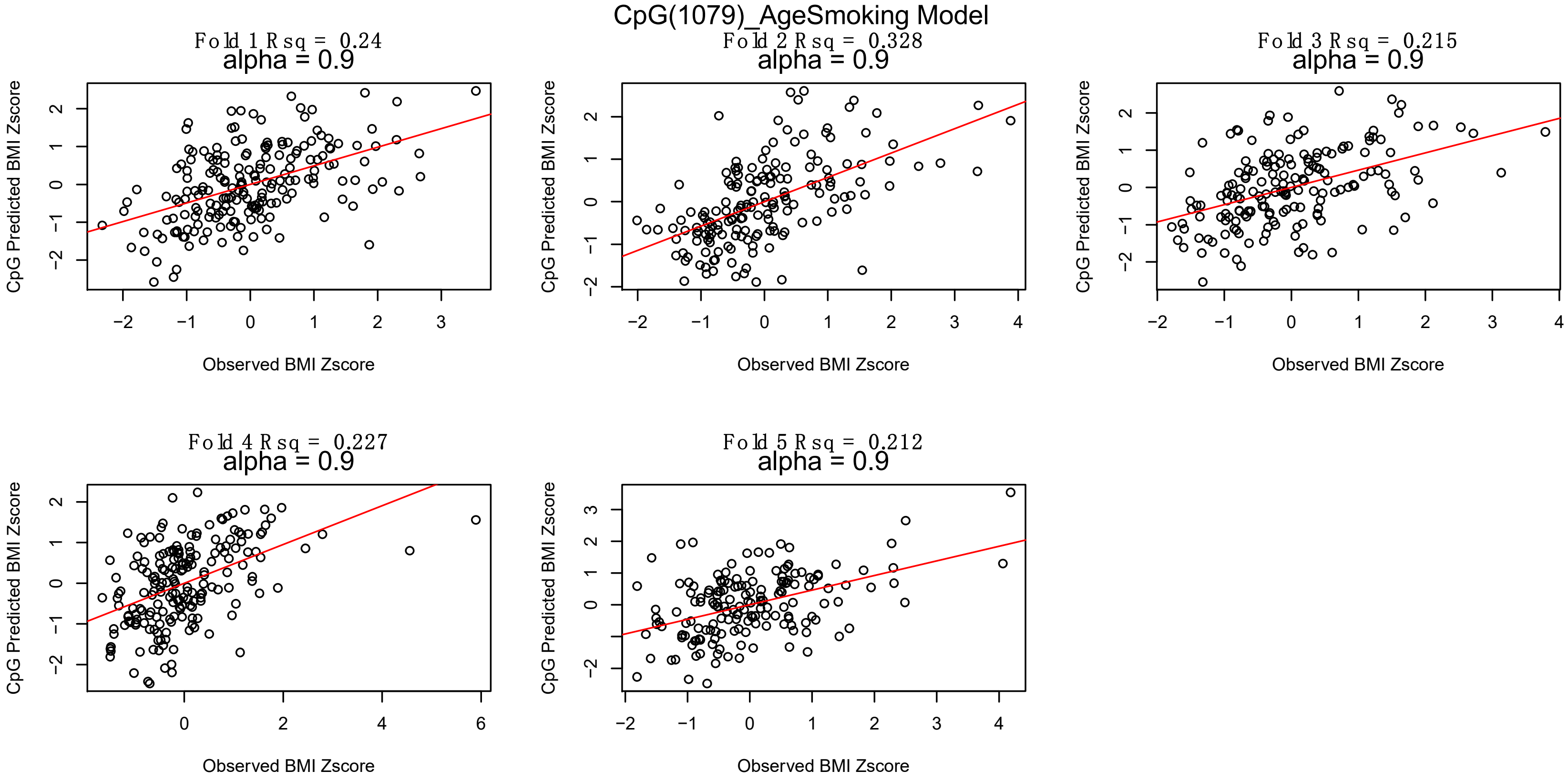

Figure S9. Cross-Validation of Predictive Accuracy for BMI Variance Using Elastic-Net Models Incorporating Age, Smoking, Viral Load, and Medication Data on CpG Sites in PWH

BMI: Body Mass Index. CpG: Cytosine-phosphate-Guanine site. MRS: Methylation Risk Score. A score that represents the cumulative effect of methylation at multiple CpG sites on a trait or disease risk, such as BMI. PWH: People With HIV. VACS: Veterans Aging Cohort Study. Rsq or R^2: Coefficient of Determination.

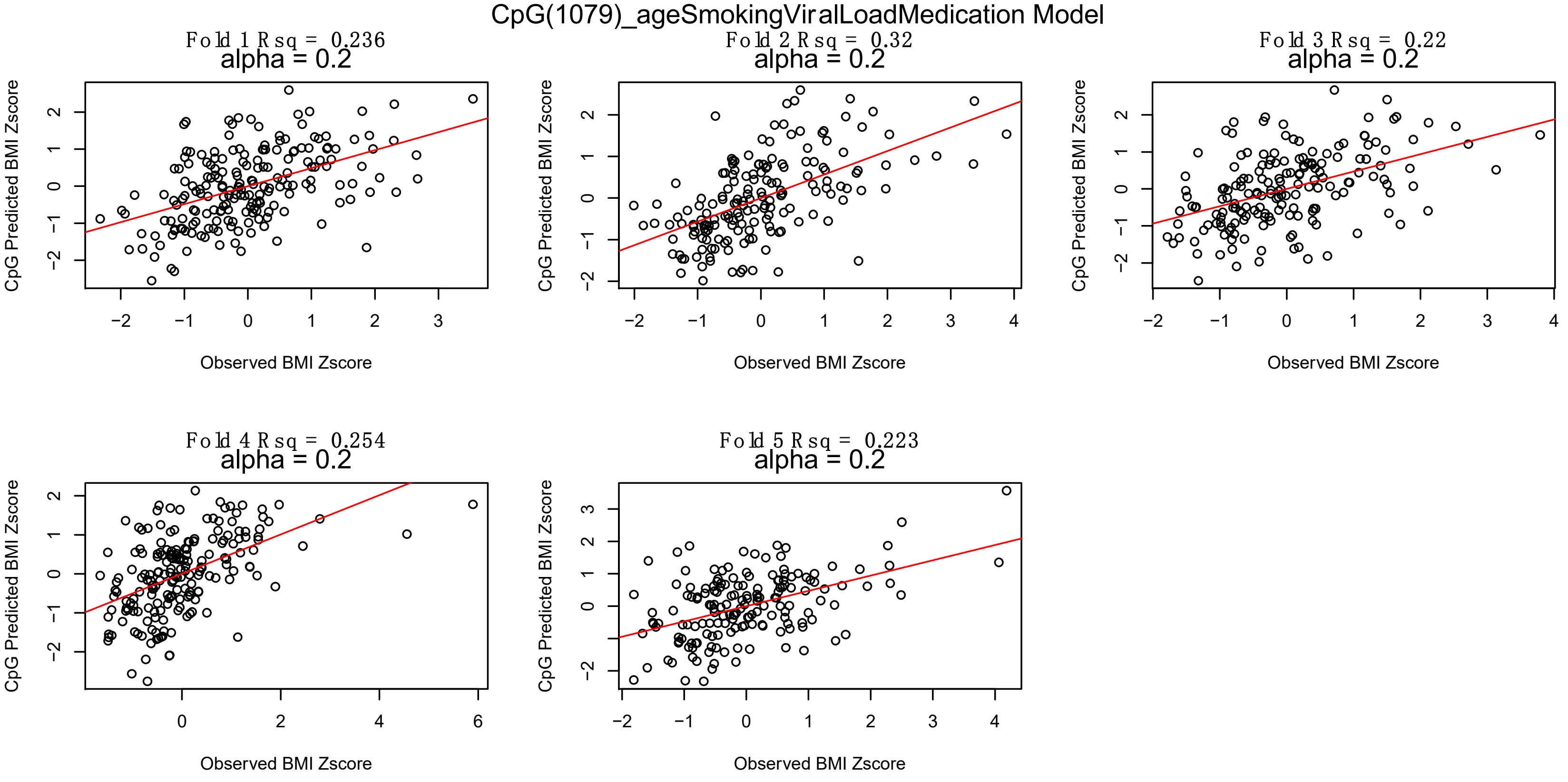

Supplement: Supplementary file 1 [file epigenomes-08-00046-s001.zip › epigenomes-3168074-supplementary.pdf]
